# The Academic Anxiety Inventory: Evidence for Dissociable Patterns of Anxiety Related to Math and Other Sources of Academic Stress

**DOI:** 10.3389/fpsyg.2018.02684

**Published:** 2019-01-15

**Authors:** Rachel G. Pizzie, David J. M. Kraemer

**Affiliations:** ^1^Department of Education, Dartmouth College, Hanover, NH, United States; ^2^Department of Psychological and Brain Sciences, Dartmouth College, Hanover, NH, United States; ^3^Department of Psychology, Georgetown University, Washington, DC, United States

**Keywords:** anxiety, math anxiety, test anxiety, trait anxiety, academic anxiety, self-report

## Abstract

Anxiety about mathematics can have detrimental effects on performance and understanding, yet little research has investigated how math anxiety is related to other types of anxiety. Here we develop the Academic Anxiety Inventory (AAI), an efficient and valid self-report measure designed to test math anxiety, as well as differentiate anxiety associated with mathematics from other contributions of anxiety across various academic domains. In Study 1, we isolated items that independently measure each domain of anxiety, reducing the overlapping variance between math anxiety and other constructs, and determining which components can or cannot be differentiated. Studies 2 and 3 demonstrate that the AAI is consistent and reliable for undergraduate and adolescent populations. In Study 3, anxiety-related performance deficits in a high school math class were associated with scores the AAI-Math subscale. In Study 4, the AAI-Math subscale was associated with perceptions of increased mathematical complexity, decreased estimations of accuracy, and increased negative emotion when participants viewed mathematical expressions. Across four studies, we demonstrate the AAI is a reliable and valid measure of math anxiety and other domains of academic anxiety, providing an efficient questionnaire to determine areas in which students may require extra support in order to reach their full potential.

## Introduction

Whereas some students flourish in stressful academic environments, many other students struggle to learn each day while dealing with various forms of anxiety. This anxiety has detrimental influences on learning and can take several forms, including anxiety associated with specific topics of study, such as mathematics, or debilitating feelings of pressure induced by testing (Alpert and Haber, 1960; Hembree, 1990). Math anxiety plays a major role in determining educational outcomes in the short-term, such as test performance or grades earned, as well as long-term outcomes such as career choices (Dew et al., 1984; Ashcraft, 2002; Beilock and Maloney, 2015). Given the significant impact of math anxiety on students, identifying the important elements that contribute to math anxiety will lead to better identification of students who may require extra support, and help us to gain understanding of how math anxiety develops over time, and to develop more effective interventions.

Here we take a data-driven approach to developing a short but useful self-report measure that separates the relevant dimension of math anxiety from other domains of academic and general anxiety. First, we evaluate whether math anxiety can be meaningfully distinguished from other domains of anxiety. We compare anxiety-related surveys across multiple conceptual frameworks in order to determine which constructs reliably emerge, and whether the data support the distinction between each separate domain of anxiety. Next, we identify a small subset of items drawn from these measures that reliably identify each separate domain of anxiety. Finally, we test the reliability and validity of our new measure in three samples of students. The following sections describe how we conceptualize math anxiety and our present approach to developing this new measure.

### Math Anxiety: Choosing a Theoretical Framework

What is math anxiety? Math anxiety refers to the feelings of stress, apprehension, and general negative affect that occurs when individuals perform mathematical computations in real-world or laboratory scenarios (Richardson and Suinn, 1972; Ashcraft, 2002), and also occurs when math anxious individuals are briefly exposed to or anticipate doing mathematics in the future (Lyons and Beilock, 2012; Pizzie and Kraemer, 2017a).

One theoretical framework useful for conceptualizing the mechanisms that lead to the negative outcomes of math anxiety draws from the biological psychology and neuroscience literature and focuses on the physiological and neural response that characterizes negative emotion. Math anxiety works on multiple levels, influencing biological responses, social attitudes including self-identification, and cognitive changes, all of which are consistent with the biopsychosocial model of negative affect (Blascovich, 1992). Math anxiety exerts changes on a biological level, such that math anxious individuals show increased activity in regions of the brain associated with threat and vigilance, and decreased neural activity in regions associated with mathematical processing (Young et al., 2012; Pizzie and Kraemer, 2017a). On a cognitive level, anxious thoughts and cognitive appraisals detract from the mathematical task at hand, and math anxiety is associated with working memory deficits (Ashcraft and Krause, 2007; Beilock, 2008). In addition to having an aversive, negative experience, stressful experiences due to anxiety also detract from performance, with math anxious individuals showing deficits in math performance (Faust et al., 1996), especially in more complex tasks or increased performance pressure (Beilock, 2008; Maloney and Beilock, 2012; Beilock and Maloney, 2015).

In addressing these sources of math anxiety, surveys that identify anxious processing across general life experience, such as the Spielberger State-Trait Anxiety Inventory (STAI; Spielberger, 2010), would be useful in indexing the levels of negative affect one experiences in general. Similarly, measures that focus on high pressure situations, such as increased performance pressure in testing environments (e.g., the Spielberger Test Anxiety Inventory, TAI; Spielberger, 2009) would also provide useful a useful quantitative assessment of these factors. Given that these processes also play a role in math anxiety, it is of theoretical and practical interest how separable are the constructs measured by these surveys from others that purport to measure math anxiety specifically. In short, we want to know, “How separable is math anxiety from anxious processing or performance pressure in general?”

Another useful theoretical framework when conceptualizing the mechanisms underlying math anxiety focuses on the social level of experience. On a social level, increased math anxiety is associated with negative perceptions of math, and gender stereotypes associated with math (Beilock et al., 2010; Gunderson et al., 2011). Indeed, sense of identity and attitudes (Oyserman et al., 2017), play a significant role in math anxiety, with many math anxious individuals identifying with the belief, “I'm not a math person,” (Schwartz, 2015). Role models such as teachers and parents likely play an important role in shaping some of these beliefs and attitudes that contribute to and compose the cognitive appraisals and worries that make up math anxiety. In particular, parents and teachers who have math anxiety are more likely to have children and students who also report increased anxiety (Beilock et al., 2010; Gunderson et al., 2011; Beilock and Maloney, 2015; Maloney et al., 2015). These social influences are reflected in attitudes about math (and other academic subjects), and are assessed by surveys that specifically query one's attitudes. In contrast to the surveys mentioned above that focus on the experience of anxious processing, these measures are focused on beliefs and attitudes, but they still provide a useful perspective on the experience of math anxiety and may predict unique variance in the negative impact of math anxiety on academic performance.

Conceptually, math anxiety encompasses physiological stress responses, negative thoughts and cognitive appraisals due to past and present experiences with math, as well as more general attitudes and stereotypes associated with math, gender, and self-identity as it relates to intelligence and academics. However, the experience of math anxiety may share a considerable amount of overlap with other experiences of anxiety or arousal—for example, the physiological measures of sympathetic nervous system activity may be relatively similar across both test anxiety and math anxiety when individuals are tasked with taking a math exam. Indeed, it may specifically be the thoughts and cognitive appraisals, stemming from memories and negative emotional experiences with mathematics that help to distinguish math anxiety from other types of anxious experience. We conceptualize the experience of math anxiety as we would any subjective emotional experience (Gross and Thompson, 2007; Gross, 2008), and evaluate the biological and physiological sensations, thoughts, memories, and appraisals, and cognitive changes that all play a role in the affect math anxious individuals experience, and that ultimately contributes to under-performance in mathematics.

### Current Approach: Data-Driven Analysis of Academic Anxiety Domains

In order to address the issue of whether math anxiety is separable from other forms of anxiety, we decided to remain agnostic as to choosing only one of the specific theoretical frameworks described above, but rather to include all of the relevant survey measures and allow the data to “speak for itself.” Our reasoning is that using this approach that includes measures of emotions, attitudes, and related experiences in a variety of contexts—academic and otherwise—allows for the most thorough assessment of which domains of anxiety are truly separable, and which are mostly collinear. Self-report questionnaires that are used to assess math anxiety (such as the Math Anxiety Rating Scale, MARS; Suinn and Winston, 2003) have difficulty separating the construct of math anxiety from anxious feelings in general (i.e., trait anxiety), and from other from other domains of anxiety, such as test anxiety (Kazelskis et al., 2000; Hopko, 2003). Therefore, studies of math anxiety have been forced to choose between providing many lengthy surveys covering anxiety in several different domains, or else risk using an impure measure of the construct they are interested in studying. Moreover, no study to date has comprehensively explored the overlap in these different, but related, anxiety constructs. It is unknown, for example, whether math anxiety can even be meaningfully separated from science anxiety (Udo et al., 2001, 2004; Bryant et al., 2012; Mallow, 2014). Therefore, in the research described here we aim to determine whether math anxiety—i.e., anxious feelings, attitudes, and performance deficits specifically associated with mathematics– exists as its own construct, separable from other related forms of anxiety. To accomplish this goal we draw from multiple existing measures that assess anxiety in each of several related domains: math anxiety, test anxiety, trait anxiety, and science anxiety. We also include in our investigation an academic domain thought to be less related to math anxiety, writing anxiety, in order to demonstrate that not all forms of academic anxiety are positively correlated. Finally, we propose and validate the Academic Anxiety Inventory (AAI), a measure of math anxiety that accurately represents this construct, as well as differentiating anxiety associated with mathematics from other contributions of anxiety across various academic domains.

Previous work has begun to characterize how math anxiety is related to broader patterns of anxious affect, including trait anxiety and test anxiety (Dew et al., 1984; Hembree, 1990; Ma, 1999; Kazelskis et al., 2000). Math anxiety and trait anxiety are moderately correlated (approximate correlation of *r* = 0.3, Daly and Miller, 1975; Betz, 1978; Daly and Wilson, 1983; Ma, 1999; Hopko, 2003), and the connection between test anxiety and math anxiety is even more robust (Kazelskis et al., 2000; Ashcraft, 2002). Test anxiety accounts for ~30% of the variance in math anxiety (Hembree, 1990) and this overlap between test anxiety and math anxiety is especially evident when examining one of the popular measures of math anxiety, the MARS (Richardson and Suinn, 1972; Suinn and Edwards, 1982; Suinn and Winston, 2003).

Richardson and Suinn (1972) created the MARS to reflect the idea that some students specifically experience anxiety in academic settings, and that this anxiety is frequently associated with mathematics. It was theorized that math anxiety was a subset of test anxiety, and this measure includes a predominance of questions regarding testing (Suinn and Edwards, 1982; Kazelskis et al., 2000). In the same vein, a subscale of one abbreviated measure of the MARS is specifically focused on anxiety about math tests (Hopko, 2003). It is not surprising, then, that correlations between the MARS (Suinn and Edwards, 1982), and measures of test anxiety generally yield strong associations, ranging from approximately *r* = 0.5 to as high as *r* = 0.8. The intercorrelations between different measures of math anxiety tend to be approximately *r* = 0.5 (Kazelskis et al., 2000), indicating that the MARS may be more strongly related to test anxiety than to other measures of math anxiety.

Although it is valuable to assess how anxiety about math tests contributes to the experience of math anxiety, this predominant focus on tests and performing mathematics in high-pressure situations may only identify a test-focused subset of highly math anxious individuals. Thus, merely asking, “How anxious do you feel about mathematics?” as a method for identifying math anxiety may also include individuals who also feel anxious about tests, or who identify as generally anxious. It is an important goal to be able to discern between different types of anxiety, and how they contribute to academic performance. These different types of anxiety (e.g., math anxiety, test anxiety, trait anxiety, and anxiety associated with writing and science) may have different causes, have different mechanisms by which they influence behavior, and may require different interventions in order to remediate the negative effects of anxiety on behavior and academic performance. For example, students who experience math anxiety only on tests might benefit from an intervention technique focused on performance pressure, such as expressive writing (Ramirez and Beilock, 2011). Conversely, math anxious students who experience a great deal of anxiety in math class every day, while doing homework, as well as on tests and quizzes, may require a different intervention in order to reverse these performance deficits and persistent negative emotions. However, if there is a large degree of overlapping variance in the popular questionnaires used to test these constructs (i.e., math anxiety and test anxiety), it might be difficult to discern the origin of these students' anxiety, as they may score similarly on individual measures of test and math anxiety. Like math anxiety, anxiety may develop around other specific academic domains, such as science anxiety (Udo et al., 2001, 2004; Bryant et al., 2012; Mallow, 2014), which is a construct that may be closely related to math or test anxiety. Writing anxiety, which is also characterized by anxiety related to a specific academic domain, likely has more distinct patterns of anxiety, and increased theoretical distance from math anxiety. In order to develop an understanding of the causes and appropriate interventions for anxiety associated with each domain, especially math, identifying specific and unique characteristics for each domain should be a priority.

In the present research, we address the following issues:

- *What is math anxiety? How is math anxiety different from other domains of anxiety?* Current measures of academic anxieties have a great deal of overlapping variance, and it is unclear if these domains of anxiety represent unique constructs, or may be representative of broader patterns of anxiety (i.e., math anxiety may be too closely related to test or trait anxiety to be considered an independent construct). In order to determine if one is math anxious, it may not be enough to ask, “How anxious do you feel about mathematics?” as this question may be answered in a similar way by math anxious students, as well as students who also experience a lot of general anxiety, as well as students who also experience anxiety on tests. It is an important goal to be able to separate anxiety associated with mathematics from other types of anxiety. Therefore, we examine the separability of these domains and aim to establish a measure of math anxiety as a unique construct.
- *Can we create a questionnaire that quickly and accurately measures math anxiety separately from other domains of anxiety?* Although separate measures exist to examine math anxiety, science anxiety, writing anxiety, test anxiety and trait anxiety, it can be fatiguing and time-consuming for participants to complete all of the relevant questionnaires in order to account for the differential influence of different kinds of anxiety. Therefore, there is a practical need for researchers and educators to assess anxiety across all these domains using a shorter survey.
- *Does this math anxiety questionnaire accurately measure anxiety and deficits associated with anxiety both in the lab and in real-world classroom environments?* Many questionnaires were developed using samples of participants that were constrained by age (e.g., only college-aged students), or were validated using only laboratory measures, and may have limited predictive validity in real-world environments. Therefore, in the present work, we test criterion validity of the newly-developed measure using performance in a high school math class.

## Study 1

In Study 1, we recruited a large sample of adults to determine whether self-report measures of math, science, writing, test and trait anxiety independently assess these constructs. In order to disentangle the experience of math anxiety from other types of anxiety, we examined self-report questionnaires that assess related general domains of test anxiety, trait anxiety, as well as anxiety specifically associated with other domains of knowledge: science and writing. Specifically, we sought to identify where the currently established measures result in overlapping constructs. Using data-driven methods, we examined participant responses across a variety of questionnaires designed to measure anxiety. In this way, we could establish if all these constructs were driven by generalized (trait) anxiety, or if anxiety that has developed around specific domains and experiences in academia (i.e., test anxiety, math anxiety, science anxiety, and writing anxiety) could be meaningfully separated. Further, we aimed to select survey questions that establish independent constructs of math, science, writing, test, and trait anxiety, reducing this overlap between constructs while maintaining a high degree of reliability. Further, in subsequent studies, we use these survey questions to create a free-standing self-report measure that is representative of mathematics and other domains of academic anxiety.

### Method

#### Participants

To validate patterns of anxiety assessed by these scales, we collected two samples from an online population, allowing us to separately analyze an original dataset for exploration as well as a holdout sample for validation of our findings in the original dataset. First we based our exploratory models on the original dataset, then we used the second sample as a separate test dataset to validate the observed patterns of anxiety associated with each academic domain.

Participants (*N* = 599) were recruited online to participate in a web-based series of questionnaires, and 34 participants were excluded from analysis, resulting in a final sample of *n* = 565 (49.0% female). An additional 143 individuals started the questionnaire task but never completed the task, and thus were not included in the datasets or samples. All participants included in the datasets completed all questions for all questionnaires. Participants were excluded from analyses on the basis of total time to complete surveys (± 3 *SD, M*_time_ = 44 min, *SD*_time_ = 21 min), and for excessively repetitive responses (<2 *SD* in variance of raw responses). Participants were recruited and compensated for their participation in this study using Amazon's Mechanical Turk (https://www.mturk.com/). Participants were residents of the United States, fluent in English, and between the ages of 18 and 82 (*M* = 35.06, *SD* = 11.03). Two-hundred eighty-five (51.6% female) subjects were included in the main dataset, and 280 subjects (50.0% female) composed the test dataset.

#### Procedure

After reviewing a consent statement and clicking to indicate their agreement, participants began the series of online surveys presented in random order. Signed written consent for this study was not required because the experiment was determined to have minimal risk, and participants read a statement reminding them about their rights and responsibilities as a participant and clicked to continue to indicate their consent. Questionnaires were presented and data collected using Qualtrics online survey platform (www.qualtrics.com). Self-report questionnaires were selected to represent several domains of academic anxiety: math anxiety, science anxiety, writing anxiety, test anxiety, and general patterns of negative affect (general/trait anxiety). To assess convergent validity, we included multiple measures for each of several academic and dispositional aspects of anxiety (see Table 1 for a complete list of measures). Participants completed all questionnaires, and scores were calculated for each of the scales and subscales listed in Table 1. Participants also provided information indicating their educational experience, age, gender, and measures of socioeconomic status at the end of the series of questionnaires. After completing the series of questionnaires, participants were given a completion code, and were compensated through Mechanical Turk. All procedures were reviewed and approved by the Dartmouth Committee for the Protection of Human Subjects (CPHS).

**Table 1:** Questionnaires selected in Study 1 to represent multiple domains of anxiety: math, science, writing, test, and trait anxiety.

	**Abbreviation**	**Questionnaire**	**Purpose**	**Example questions**	**Number of citations**
Math anxiety	MARS	Math Anxiety Rating Scale (Richardson and Suinn, 1972; Suinn and Edwards, 1982; Suinn et al., 1988; Hopko, 2003; Suinn and Winston, 2003)	30 items. Negative attitudes toward math and math tests	“Taking an examination (final) in a mathematics course”	1,023^*^
	MAS	Fennema-Sherman Mathematics Attitudes Scale (Mulhern and Rae, 1998)	108 items. Negative attitudes regarding math motivation including self-perception, teachers, parents	“I am sure that I can learn mathematics”	1,016
	MAT	Mathematics Attitude Scale (Alken, 1974)—Enjoyment and Value subscales	22 items. Positive attitudes toward mathematics	“Mathematics helps develop a person's mind and teaches him to think”	232
Science anxiety	SAQ	Science Anxiety Questionnaire (Udo et al., 2001, 2004; Bryant et al., 2012; Mallow, 2014)	44 items. Negative attitudes toward science and non-science activities	“Memorizing the names of the elements in the periodic table”	132^*^
	SMQ	Science Motivation Questionnaire (SMQ; Glynn et al., 2009)	30 items. Positive Attitudes toward science	“The science I learn is more important to me than the grade I receive”	125
Test anxiety	TAI	Spielberger Test Anxiety Inventory (Spielberger, 2009)	20 items. Negative attitudes toward testing environments and anticipation	“I freeze up on important exams”	726
	STABS	Suinn Test Anxiety Behavior Scale (Suinn, 1969; Ginter et al., 1982)	50 items. Negative attitudes toward testing, anticipation, and outcomes of testing	“Thinking about a coming exam the night before its scheduled date”	119
Writing anxiety	WA	Daly-Miller Test of Writing Apprehension (Daly and Miller, 1975; Daly and Wilson, 1983)	26 items. Positive attitudes toward writing process and evaluation of writing	“I like seeing my thoughts on paper”	165
	BWA	Blake's “The Attitude Scale: Writers and Writing,” (Blake, 1975)	20 items. Positive attitudes toward writing practices and perception of authors	“I like to write something every day”	5
Trait anxiety	STAI	Spielberger State/Trait Anxiety Inventory—Trait subscale (Spielberger, 2010)	20 items (subscale). Trait subscale represents broader patterns of anxiety and uneasiness	“I worry too much over something that really doesn't matter”	22,785
	PANAS—PA, PANAS—NA	Positive (PANAS-PA) and Negative Affect Schedule (PANAS-NA); (Watson et al., 1988)	20 items. Rate positive (PA) or negative (NA) trait/emotion words based on how general feelings	Positive: “interested,” “excited” Negative: “guilty,” “upset”	19,344
	EXT, NEU	Extroversion (EXT) and Neuroticism (NEU) subscales of the NEO (Costa and McCrae, 1985; Costa et al., 1991)	24 items. Tendency to feel energized and active (EXT), or anxious and discouraged (NEU)	“I laugh easily” (EXT), “I often feel tense and jittery” (NEU)	1,076

*Number of citations was estimated using number of citations for the questionnaire using Google Scholar in November 2015*.

* *Citations for multiple versions of the questionnaire (e.g., the MARS and MARS-A for adolescents), or multiple citations that included the text of the questionnaire were summed to estimate the number of citations*.

### Results

#### Analyses

Statistical procedures were completed using a combination of R statistical software (v. 0.98.978; http://www.R-project.org) and SPSS (IBM Corporation). The goals of this study were: (a) to examine whether the current questionnaires independently represent the domains of math, science, writing, test, and trait anxiety, (b) to identify areas of possible overlap, and (c) to isolate components of each construct that reduce this overlapping variance, identifying questions that best represent these domains of anxiety. Instead of using methods that might introduce experimenter bias into the selection of items, for example, only selecting items that we hypothesized to best represent what we theoretically believe to represent math anxiety, we used data-driven methods to assess the overlap between different domains and questionnaires. From there, we could select the items that best represent each unique domain of anxiety, reducing overlap between domains of anxiety, and ideally, identifying questions that uniquely represent each domain of anxiety. To accomplish these goals, we first assessed overlap between domains using bivariate correlations between existing questionnaires. We then used principal component analysis (PCA) to determine whether the hypothesized five-domain structure would emerge. Then we used factor analysis with the scores from each scale (or subscale) to determine whether each scale would load onto the hypothesized domain. Finally, we used item-wise PCA with all the items from all the scales to identify the questions that best represent the unique aspects of each dimension. Once these independent components were isolated, we were then able to choose ten items from each domain to generate a new survey for evaluation.

#### Bivariate Correlations

Each questionnaire was scored by calculating the average response for each scale or subscale (reverse-scored items were reversed before mean response was calculated). Bivariate correlations were used to explore the relationships between each questionnaire (Table 2; for regression models for each domain, see Supplementary Material). Within these results, we observe a moderate to high degree of correlation between the questionnaires assessing each domain across both samples. For example, the TAI and STABS (both measures of test anxiety; see Table 1 for description of questionnaires), are highly correlated across both samples (*r* = 0.74). Moreover, both of these test anxiety measures are less correlated with measures of trait anxiety (*r* = 0.34 −0.45) than they are correlated with each other. Overall, the pattern of correlations indicates a higher degree of overlap within each domain than between domains. However, the few exceptions in which there are large amounts of overlapping variance between domains (e.g., the MARS has higher correlations with measures of test anxiety than other measures of math anxiety) indicate that there are instances in which existing questionnaires may not be representative of the domain purportedly tested.

**Table 2:** Bivariate Pearson correlations between self-report measures of academic anxieties, general anxiety, and affect in Study 1.

		**1**	**2**	**3**	**4**	**5**	**6**	**7**	**8**	**9**	**10**	**11**	**12**	**13**	**14**	**15**
Domain-general anxiety and affect	1. PANAS-NA	–	**−0.14**	***−0.37***	***0.68***	***0.72***	***0.48***	***0.46***	***−0.21***	***0.51***	***0.53***	***−0.25***	***0.24***	***0.46***	***−0.19***	0.01
	2. PANAS-PA^*^	***−0.27***	–	***0.55***	***−0.48***	***−0.48***	−0.10	0.02	***0.34***	−0.04	−0.05	***0.37***	***−0.39***	−0.09	***0.24***	***0.17***
	3. NEO-EXT^*^	***−0.43***	***0.62***	–	***−0.63***	***−0.65***	***−0.27***	***−0.20***	***0.25***	***−0.21***	***−0.25***	***0.30***	***−0.35***	***−0.24***	***0.28***	**0.14**
	4. NEO-NEU	***0.67***	***−0.52***	***−0.61***	–	***0.90***	***0.47***	***0.45***	***−0.36***	***0.43***	***0.41***	***−0.35***	***0.38***	***0.50***	***−0.25***	−0.05
	5. STAI	***0.66***	***−0.56***	***−0.67***	***0.87***	–	***0.50***	***0.45***	***−0.33***	***0.43***	***0.44***	***−0.36***	***0.38***	***0.50***	***−0.26***	−0.03
Test anxiety	6. TAI	***0.40***	**−0.15**	***−0.20***	***0.45***	***0.41***	–	***0.74***	***−0.28***	***0.62***	***0.59***	***−0.33***	***0.32***	***0.71***	***−0.30***	−0.08
	7. STABS	***0.36***	0.01	−0.11	***0.36***	***0.34***	***0.74***	–	***−0.23***	***0.67***	***0.64***	***−0.28***	***0.25***	***0.73***	***−0.28***	−0.06
Science anxiety	8. SMQ^*^	**−0.13**	***0.26***	***0.17***	***−0.25***	***−0.23***	**−0.14**	−0.11	–	***−0.40***	***−0.28***	***0.51***	***−0.47***	***−0.38***	**0.16**	**0.18**
	9. SAQ-S	***0.33***	0.00	−0.05	***0.32***	***0.28***	***0.44***	***0.64***	***−0.33***	–	***0.88***	***−0.30***	***0.30***	***0.68***	***−0.23***	**−0.12**
	10. SAQ- NS	***0.32***	0.01	−0.07	***0.28***	***0.27***	***0.40***	***0.59***	***−0.22***	***0.86***	–	***−0.20***	***0.23***	***0.60***	***−0.35***	***−0.20***
Math anxiety	11. MAT^*^	**−0.12**	0.08	0.10	***−0.23***	***−0.18***	***−0.34***	***−0.28***	***0.39***	***−0.30***	***−0.19***	–	***−0.88***	***−0.53***	**0.12**	0.08
	12. MAS	***0.19***	**–*****0.18***	**−0.16**	***0.32***	***0.27***	***0.39***	***0.35***	***−0.39***	***0.37***	***0.25***	***−0.85***	–	***0.51***	−0.11	−0.07
	13. MARS	***0.37***	0.00	**−0.12**	***0.36***	***0.34***	***0.62***	***0.74***	***−0.18***	***0.64***	***0.57***	***−0.51***	***0.58***	–	***−0.20***	−0.02
Writing anxiety	14. WA^*^	**−0.14**	***0.23***	***0.20***	**−0.16**	**−0.17**	***−0.20***	**−0.15**	**0.15**	**−0.12**	***−0.29***	−0.03	0.03	−0.03	–	***0.66***
	15. BWA^*^	−0.03	**0.16**	**0.12**	−0.04	−0.07	−0.05	−0.03	**0.16**	−0.05	***−0.22***	−0.03	0.00	0.02	***0.75***	–

*Correlations listed in bold are significant at α = 0.05, items significant at α = 0.01 are listed in bold italics. Data from the original dataset is presented below the diagonal (N = 285). Data from the test dataset is presented above the diagonal (N = 280). Shaded regions represent correlations from each dataset that occur within the same domain of anxiety (i.e., measures of test anxiety correlated with other measures of test anxiety). For abbreviations, see Table 1*.

* *Higher scores on scale represent more positive attitudes*.

#### Identifying Number of Domains With PCA

In order to determine the number of domains represented by these data, we conducted a PCA (unrotated) using the total scores from the scales and subscales hypothesized to represent math, science, writing, test and trait anxiety (within the main dataset, *N* = 285). Overall, we find 15 components represent 100% of the variance in these data. The first 5 components represent ~81% of the variance, and standard deviation for each of these components is ~1 (PC 1: *SD* = 2.32, Proportion of Variance (POV) = 0.36, PC 2: *SD* = 1.58, POV = 0.17, PC 3: *SD* = 1.35, POV = 0.12, PC 4: *SD* = 1.24, POV = 0.10, PC 5: *SD* = 0.95, POV = 0.06). For the other additional components in this analysis, the standard deviation continues to fall below 1, and the proportion variance accounted for begins to asymptote after the first 5 components. Here these results indicate that five factors best represent the data (after the first five factors the SD continues to asymptote). Indeed, it could have been the case that fewer components could have represented the data, perhaps indicating that multiple domains of anxiety would be driven by more broad patterns of anxiety (i.e., we could have just found 2 factors, representing trait and test anxiety, with the other academic domains being represented therein). Instead, five components were selected to best represent the variance in these data, though further factor analysis was needed to interpret the underlying structure of these data.

#### Confirming Domain Structure With Factor Analysis

**Figure 1:**
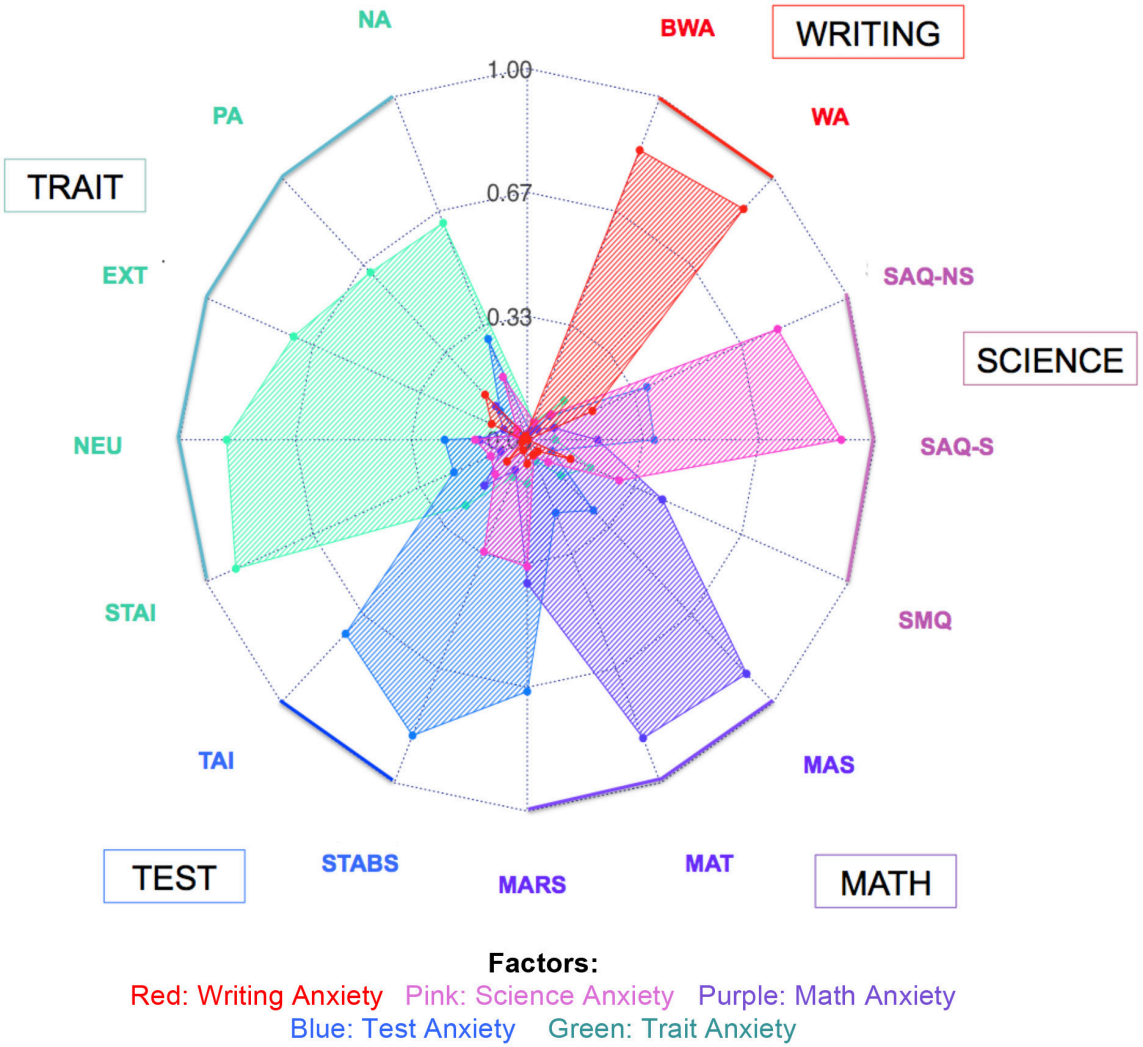
Varimax-rotated factor analysis using single scales in Study 1. Factor scores for each factor are represented for writing, science, math, test, and trait anxiety in the main sample (*N* = 285). Abbreviations for each scale are listed around the circle. For scale abbreviations, see Table 1. Colors represent the hypothesized domains for each scale (e.g., the MARS is hypothesized to represent math anxiety, the TAI hypothesized to represent test anxiety). Values on the radar plot represent absolute values of the factor loadings for each scale. Zero is represented at the center of the plot, with increasing factor scores radiating out from the center. Values closer to the outside of the plot indicate higher factor loadings.

Figure 1 depicts a maximum-likelihood factor analysis (MLFA) with varimax rotation using the scores from the existing scales to establish whether the latent factors that emerged from this analysis would align with the hypothesized five-domain structure (math, science, writing, test, and trait). We find that the five factor structure is sufficient to represent these data, *X*^2^_(40)_ = 90.1, *p* < 0.001 [Tucker-Lewis Index of factoring reliability: 0.954; RMSEA index = 0.068 (95% CI: 0.048–0.085); BIC: −135.96]. As hypothesized, the factor analysis using five factors largely overlaps with the hypothesized domains, such that the latent factors are representative of math, science, writing, test, and trait anxiety—as the majority of these existing scales in each domain have high loadings on the appropriate factor. That these factors are independently represented by the scales is an important distinction. These data could have been represented by fewer factors, perhaps with broader patterns of anxiety representing responses across domains of anxiety. For example, it might have been the case that we only found two factors representing anxiety: a factor representing test, math, and quantitative/science anxiety, and another factor representing trait/general anxiety as well as writing anxiety. A result such as this would indicate that math anxiety might not be a unique or independent construct, but that math anxiety would merely be an instance of test anxiety associated with a quantitative measure. However, from this factor analysis (Figure 1), we observe that the scales represent 5 independent domains of anxiety: math anxiety, science anxiety, writing anxiety, test anxiety, and trait anxiety. The current measures of these domains do sufficiently separate anxiety into unique domains that can be separately measured.

Although we can separate each domain using multiple measures, in looking within the results reflected for each scale, there are some important ways in which individual scales represent overlap between domains. In other words, although the domains of anxiety are separable using multiple measures of anxiety, individual scales that are hypothesized to represent a specific domain may in fact be more closely associated with a different domain. As seen in Figure 1, the results of this factor analysis demonstrate the overlap between math, test, and science anxiety, in that several existing scales purported to measure one domain seem to be strongly associated with other domains. For example, the science domain seems to be largely driven by both science and non-science subscales of the SAQ. The SMQ, which is also hypothesized to assess science anxiety, has a relatively low loading on the science factor, and instead has a higher loading on the factor associated with math. Similarly, the MARS, a common scale used to assess math anxiety, has a stronger relationship with the factor representing test anxiety than it does with the factor representing math anxiety. Overall, the results of this factor analysis reveal that the domains of math, science, writing, test, and trait anxiety correspond to separable latent factors. However, using the existing questionnaires to test these domains does not represent the optimal method for identifying each domain of anxiety due to several of the scales loading onto an unintended factor.

#### Development of a New Measure

To better assess these domains of math, science, test, and trait anxiety, while attempting to decrease the overlap between these domains, we developed a new survey by selecting the items that best represent these constructs from the existing scales. In order to select these items, we conducted a varimax-rotated PCA using the individual items from each existing scale (Table 3). Using data from the main dataset (*N* = 285), the response to each individual question was scaled from 0 to 1 to eliminate the problem of different scale endpoints. In order to represent attitudes in a consistent manner, some items were reversed such that greater scores represent attitudes consistently across all of the scales within each domain. As a result, for all domains of anxiety, except for writing anxiety, greater scores reflect negative attitudes and feelings. For writing anxiety, greater scores reflect positive attitudes and feelings toward writing.

**Table 3:** Items identified by item-wise PCA in Study 1 for each of 5 principal components: test, math, science/quantitative, trait, and writing.

**Factor**	**Survey**	**Item**	**PC 1**	**PC 2**	**PC 3**	**PC 4**	**PC 5**
Test anxiety	STABS	Having a test returned	**0.77**	0.08	0.01	0.07	−0.01
	STABS	Being in class waiting for my corrected test to be returned	**0.82**	0.15	0.21	0.07	−0.06
	STABS	Studying for a test the night before.	**0.79**	0.05	0.05	0.14	0.00
	STABS	Waiting for a test to be handed out	**0.79**	0.06	0.03	0.08	0.05
	STABS	Waiting to see my letter grade on the test	**0.81**	0.06	−0.09	0.07	0.07
	STABS	Studying for a midterm	**0.79**	0.08	0.11	0.05	−0.01
	STABS	Studying for a final	**0.80**	0.11	0.09	0.07	0.00
	STABS	Reviewing study materials the night before an exam	**0.81**	0.16	0.07	0.02	−0.01
	STABS	Thinking about a coming exam the night before its scheduled date	**0.85**	0.05	−0.13	0.10	0.04
	STABS	Thinking about a coming exam the hour before its scheduled date	**0.77**	0.11	0.09	0.05	−0.03
Math anxiety/attitudes	MAT	Mathematics is less important to people than art or literature	−0.34	**−0.75**	0.03	0.07	−0.06
	MAT	Mathematics helps develop a person's mind and teaches him to think	−0.26	**−0.79**	0.16	0.03	−0.07
	MAT	Mathematics is very interesting, and I have usually enjoyed courses in this subject	−0.24	**−0.80**	0.10	0.01	−0.10
	MAS	I am sure I could do advanced work in mathematics [R]	0.24	**0.76**	0.01	0.11	0.02
	MAS	I think I could handle more difficult mathematics [R]	0.27	**0.78**	−0.04	0.03	0.00
	MAS	I'm not good at math	0.34	**0.76**	−0.06	0.02	0.06
	MAS	For some reason even though I study, math seems unusually hard for me	0.33	**0.75**	−0.07	0.01	0.07
	MAS	Mathematics is enjoyable and stimulating to me [R]	0.25	**0.82**	−0.05	−0.06	0.07
	MAS	Figuring out mathematical problems does not appeal to me	0.26	**0.77**	−0.06	0.02	0.01
	MAS	I do as little math a possible	0.28	**0.80**	0.07	0.01	0.05
Quantitative/Science anxiety	SAQ-S	Cooling down a hot tub of water to an appropriate temperature for a bath [NS]	0.21	0.08	**0.70**	−0.01	0.00
	SAQ-NS	Focusing the lens on your camera [NS]	0.14	0.03	**0.70**	0.03	−0.04
	SAQ-S	Using a thermometer in order to record the boiling point of a heating solution	0.19	0.05	**0.67**	0.09	0.05
	SAQ-NS	You want to vote on an upcoming referendum on student activities fees, and you are reading about it so that you might make an informed choice [NS]	0.27	−0.03	**0.62**	0.04	−0.10
	SAQ-NS	Filling your bicycle tires with the right amount of air [NS]	0.23	0.00	**0.62**	0.09	−0.03
	SAQ-S	Mixing boiling water and ice to get water at 70 degrees Fahrenheit	0.24	0.07	**0.62**	0.04	0.07
	SAQ-S	Focusing a microscope	0.18	0.08	**0.65**	0.09	0.03
	MAS	Studying mathematics is just as appropriate for women as for men [R]	−0.10	0.16	**0.64**	−0.06	−0.08
	MARS	Adding 976 + 777 on paper	0.31	0.16	**0.63**	0.02	−0.04
	MARS	Reading a cash register receipt	0.26	0.11	**0.64**	0.04	−0.07
Trait anxiety	NEO-EXT	I am not a cheerful optimist [R]	−0.11	−0.12	0.08	**−0.66**	0.03
	NEO-EXT	I am a cheerful, high-spirited person	−0.05	−0.07	0.14	**−0.69**	0.08
	NEO-NEU	Sometimes I feel completely worthless	0.23	0.05	−0.01	**0.71**	−0.02
	NEO-NEU	Too often, when things go wrong, I get discouraged and feel like giving up	0.23	0.21	−0.02	**0.72**	−0.07
	STAI	I feel pleasant [R]	0.01	0.10	0.02	**0.72**	−0.08
	STAI	I feel like crying	0.15	0.07	−0.03	**0.70**	−0.08
	STAI	I wish I could be as happy as others seem to be	0.06	0.04	−0.08	**0.75**	−0.07
	STAI	I am losing out on things because I can't make up my mind soon enough	0.05	0.10	0.03	**0.66**	−0.06
	STAI	I feel rested [R]	0.04	0.08	0.07	**0.67**	−0.02
	STAI	Some important thought runs through my mind and bothers me	0.14	0.08	0.00	**0.74**	0.03
Writing anxiety	WA	I look forward to writing down my ideas [R]	−0.02	0.06	−0.01	0.01	**0.74**
	WA	I am afraid of writing essays when I know they will be evaluated [R]	−0.07	0.03	−0.17	−0.14	**0.76**
	WA	Handing in a composition makes me feel good [R]	0.00	0.11	−0.04	−0.11	**0.78**
	WA	Expressing ideas through writing seems to be a waste of time.	−0.06	0.08	0.01	−0.14	**0.74**
	WA	Writing is a lot of fun [R]	0.00	0.08	0.03	−0.09	**0.80**
	WA	I like seeing my thoughts on paper [R]	0.09	0.03	0.00	−0.10	**0.81**
	WA	Discussing my writing with others is enjoyable [R]	0.03	0.02	−0.08	−0.11	**0.79**
	WA	I have a terrible time organizing my ideas in a composition course	−0.02	0.09	0.00	−0.09	**0.84**
	WA	When I hand in a composition, I know I'm going do to poorly	−0.08	0.02	−0.04	−0.07	**0.79**
	WA	I don't like my compositions to be evaluated	−0.05	0.09	−0.06	−0.03	**0.77**

*Ten items with highest loadings for each of five components (PC1: Test, PC2: Math, PC3: Science/Precision. PC4: Trait, PC5: Writing) identified by varimax-rotated PCA with all items (data from main sample, N = 285). These items were used to construct the 10-item reduced component scales. [R] indicates that item is reverse-scored in the original scale. PCA loadings >0.5 depicted in bold to illustrate items that have high loadings on a particular component*.

**Figure 2:**
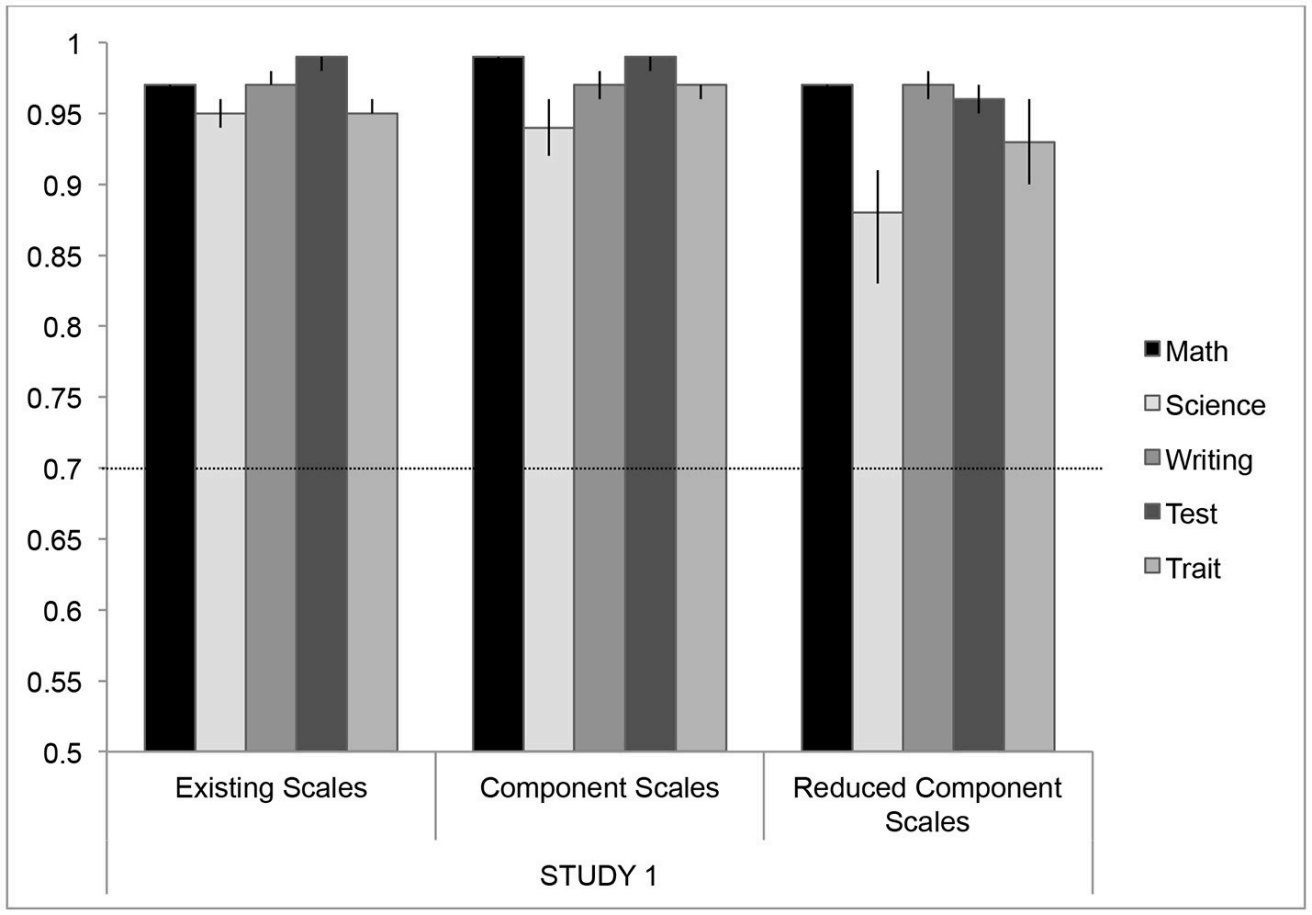
Cronbach's Alpha values for each domain of anxiety across different measures in test dataset (*N* = 280) in Study 1. Error bars represent bootstrapped 95% confidence intervals (CIs; these error bars represent 1,000 randomized iterations of the data). Some of these 95% CIs represent values that are close to the ceiling of Cronbach's alpha values, and so the bootstrapped CI reaches an asymptote. For some of these subscales, the 95% CIs may also be asymmetrical as the randomized bootstrapping reached lower alpha values, representing more variability in those subscales. The dashed line indicates the recommended criteria for adequate reliability using Cronbach's Alpha (Peterson, 1994).

The main sample (*N* = 285) was used to calculate the item-wise PCA (Table 3), while the test sample (*N* = 280) was used to evaluate internal consistency (Figure 2) and the degree of overlap between the components identified by the item-wise PCA (Table 4). Each of the components comprised a different number of items, so we also constructed a “reduced” component score for each domain by selecting the ten items that had the greatest factor coefficients. In this way, we were able to evaluate how each component was related, as well as evaluating whether a reduced number of items would have similar consistency in each domain of anxiety.

**Table 4:** Correlations between each domain of academic anxiety: math, science, writing, test, and trait for Study 1.

**Single scales**						**Component scales**						**Reduced component scales**					
	**STAI**	**TAI**	**SAQ-S**	**MARS**	**WA**		**Trait**	**Test**	**Science**	**Math**	**Writing**		**Trait**	**Test**	**Science**	**Math**	**Writing**
STAI	***–***	***0.50***	***0.43***	***0.50***	***−0.26***	Trait	***–***	***0.57***	***0.35***	***0.32***	**−0.16**	Trait	***–***	***0.31***	***0.21***	***0.31***	−0.11
TAI		**–**	***0.62***	***0.71***	***−0.41***	Test		***–***	***0.48***	***0.41***	***−0.25***	Test		***–***	***0.36***	***0.30***	**−0.17**
SAQ-S			–	***0.68***	***−0.23***	Science			***–***	***0.25***	***−0.22***	Science			***–***	***0.15***	**−0.16**
MARS				***–***	***−0.20***	Math				***–***	−0.05	Math				***–***	−0.02
WA^*^					**–**	Writing					***–***	Writing					***–***

*All correlations were calculated from mean scores in the test dataset (N = 280). Pearson correlation coefficients are represented above the diagonal. Correlations listed in bold are significant at α = 0.05, items significant at α = 0.01 are listed in bold italics. Higher scores in trait, test, science, and math anxiety represent more negative attitudes. Higher scores in writing anxiety represent more positive attitudes*.

As hypothesized, five components were identified by the item-wise varimax-rotated PCA, which accounted for 42% of the variance, and the fit based upon off-diagonal values = 0.94. From the items identified by each component, we interpret these components (Table 3) apparently representing test anxiety, math anxiety/attitudes, quantitative or science anxiety, writing anxiety, and trait anxiety. Each component included items from a variety of single scales, usually within the same *a priori* domain.

However, in some cases, items from one purported domain (i.e., items from the MARS) were associated with another domain strongly enough that they were included in the reduced set of items for a different domain (i.e., test anxiety). Again, this illustrates that the MARS may be more appropriate for evaluating test anxiety, although it is widely thought to represent self-reported math anxiety. Factor 3 also illustrates that there is also a great deal of overlap between science and math items in the quantitative or science-related component. Indeed, because the majority of the questions in this factor are drawn from the Science Anxiety Questionnaire (Mallow, 2010, 2014; Mallow et al., 2010), including questions drawn from the “science” subscale, the “non-science” subscale, we interpret that this line of questions is associated with quantitative understanding and tasks that require precision, such as science and math. As we observe in other domains (e.g., the MARS and test anxiety), a couple items from math questionnaires also load onto this factor. As such, we refer to this domain as quantitative/science anxiety. Though this may seem to be strongly associated with behaviors that may relate to math anxiety, we believe that this factor likely represents quantitative tasks that are less specific to math anxiety and more related to anxiety related to precision and quantitative knowledge that would be utilized in the science and general quantitative domain.

In the questions selected for this math component, the majority of these questions (and their subsequent wording for Studies 2–4) are drawn for the MAS—the “Mathematics Attitudes Scale” (see Tables 1, 3). These questions are drawn from a scale designed to assess multiple facets of math anxiety, including attitudes. As we understand the experience of math anxiety, it may be the specific cognitions and reappraisals that define math anxiety as separate from other experiences of anxiety. For example, participants who strongly endorse “feeling anxious on a math test” may also experience a lot of test anxiety, or just a lot of anxiety in general. As such, questions that simply ask about feeling anxious may not have enough specificity to identify the unique aspects of math anxiety. Indeed, it is interesting that the questions that seem to represent the most unique aspects of math anxiety are those that represent attitudes, and as is frequently the case, questions that are worded to represent positive aspects or attitudes toward mathematics. When these questions are analyzed, they are “reverse scored” to represent more negative attitudes, such that even though these questions are worded in a positive direction, the scores ultimately represent more negative attitudes. As it is commonly socially acceptable to espouse negative beliefs toward mathematics (many people might identify with the statement, “I'm not a math person,” etc.), utilizing questions that ask about expression of positive attitudes may identify a more accurate and diagnostic range of math anxiety when participants answer these questions. We would argue that these cognitions and attitudes make up an important aspect of math anxiety, and can still be accurately called math anxiety.

To test these components against the existing scales, we used the items identified by the item-wise PCA and calculated mean responses for each component and each reduced component using the test dataset (*N* = 280), and we compared them to the popular existing scales measuring each domain (Figure 2). Cronbach's alpha was calculated for each of five domains for the existing scales (the most popular scale was taken as representative for each domain; see Table 1), component scales, and reduced component scales, which used the 10 most representative items from each component (Figure 2). General criteria for a high degree of reliability suggest alpha values above 0.7 are highly reliable (Nunnally and Bernstein, 1978; Peterson, 1994). Overall, reliability for all the scales is extremely high—generally above 0.9—and this is maintained across the measurements for each domain. While there is a slight drop in reliability for the reduced component scales (10-items) in science anxiety (α = 0.88) and trait anxiety (α = 0.93), these values are still well above the suggested criteria for reliability within a scale.

Ultimately, the goal of creating these reduced component scales was to maintain the internal consistency within each domain, while reducing the amount of overlap between these domains. To evaluate this overlap within the test data, we calculated Pearson bivariate correlations between the domains of academic anxiety for the existing single scales, the component scales, and the reduced component scales (Table 4). Whereas, the single scales exhibit a great deal of overlap between domains, the correlations between the domains of academic anxiety are attenuated when using component scores. The reduced component scales (using only 10 items from the PCA, see Table 3 for list of items) reduce this overlap even further, such that the correlations between the domains are reduced by almost half when compared to the single scales (Table 4). Overall, the reduced component scales maintain a high degree of internal consistency (Figure 2) while reducing the overlap between these domains (Table 4).

### Discussion

In Study 1, we sought to explore the commonly-used measures of math, science, and writing anxiety, illustrating the relationships between these domains and their association with test anxiety and trait anxiety. While many of these domains of academic anxiety have a high degree of overlap, our goal in this analysis was to examine the current measures of these domains of anxiety as they relate to math anxiety, identify the overlap in these questionnaires, and isolate independent aspects of math, science, writing, test, and trait anxiety, in order to more accurately assess each as a unique construct and to separate these components from math anxiety.

In exploring how the popular measures in each of these domains are related using correlation and factor analysis, we found strong associations between math, science, and test anxiety. Consistent with previous literature (Hembree, 1990; Kazelskis et al., 2000), we found that the MARS has a strong relationship with test anxiety and trait anxiety, and may be more representative of test anxiety than math anxiety, *per se*. The results of a factor analysis using existing questionnaires demonstrate that although some scales span domains, we isolated five unique domains that emerge from the popular measures, representing math, quantitative/science or “precision,” writing, test, and trait anxiety. This result, as well as other instances of overlap between domains of anxiety, highlights a need for an improved tool for assessing self-reported math anxiety that allows identification of anxiety in this and other academic domains while reducing the contamination between them.

Study 1 uses data-driven methods to test the idea that there may be a substantial amount of overlap in the ways that we currently test for math anxiety and other types of anxiety. From this perspective, we question the idea that one can just ask a student or participant, “How anxious do you feel about math?” and get an accurate measure of math anxiety that's would not also be answered in a similar way by someone who feels a great deal of anxiety about tests, or a great deal of general anxiety. Because interviews and questionnaires can be biased based on the perspective of the experimenter or researcher, here we used experimental methods that utilize response patterns of the participants to drive the conceptualization and understanding of the unique aspects of math anxiety that are separate from other domains of anxiety.

Principal component analysis allowed us to examine how the five academic anxiety factors manifested across specific questions within the existing scales. We identified a set of 50 items drawn from these questionnaires that uniquely represent anxiety associated with math, science/precision, writing, taking tests, and general patterns of anxious affect (trait anxiety). Reducing the number of questions from ~500 to 50 would greatly decrease the amount of time required to assess math anxiety while accounting for the separate contributions from these other academic anxiety constructs. For students who have limited time during a school day or limited time to participate in an experimental session, completing extra questionnaires may not be feasible (e.g., adding an extra 45 min of questionnaires would represent a significant period of time during a school day). Survey response fatigue has been shown to influence the quality of data, both by negatively influencing the quality of the data collected (Egleston et al., 2011), and suggesting that although it may depend on the survey content, it is frequently the case that shorter surveys are preferable for maintaining appropriate response rates (Rolstad et al., 2011).

Using these new component scales with only 10 items per domain allows us to efficiently assess each domain of academic anxiety with a similarly high degree of reliability to the original scales, while reducing the overlap between the domains of math, science, writing, test and trait anxiety. Study 1 demonstrated that these items represent a reliable measure to assess math anxiety with stable and unique domains to account for the additional factors that may be related to academic anxiety. We use these items to generate a new inventory to assess math anxiety and other academic anxieties, the AAI, validated in the subsequent studies.

## Study 2

Study 1 established that a set of questions could be derived from current measures of math, science, writing, test and trait anxiety that effectively measure math anxiety, and account for academic anxiety in other domains of science/precision, writing, test, and trait anxiety. Moreover, these items maintain a high degree of reliability while reducing the overlap between these domains. We distilled these items into a new scale we have called the AAI (Please see Appendix for AAI Questionnaire and Scoring guide). In Study 2, we evaluated the reliability of the AAI in undergraduates, assessed the degree to which the AAI was correlated with other measures of math and trait anxiety, and evaluated whether the factor structure would again replicate the same five factor structure across the 50 items included in the AAI.

### Method

#### Participants

Two-hundred and forty-eight undergraduates from introductory psychology and neuroscience classes completed the AAI, STAI (trait), and MARS (math). Eighteen students were enrolled across 2 terms and were invited to take the AAI twice, 16 students had scores for both terms. For students with duplicate scores, these items and scores were averaged across both terms. The resulting sample of 236 undergraduate students was 69% female, ranged in age from 18 to 24 years of age, with an average age of 19.36 years (*SD*_age_ = 1.19).

#### Procedure

Participants were invited to complete a variety of questionnaires online for course extra credit, and were informed that these questionnaires would be utilized for research purposes. All participants reviewed a consent statement and clicked to indicate their agreement. Signed written consent for this study was not required because the experiment was determined to have minimal risk, and participants read a statement reminding them about their rights and responsibilities as a participant and clicked to continue to indicate their consent. All procedures were approved by the CPHS.

### Results

#### Reliability

We assessed test-retest reliability in a small subsample of these participants (*N* = 18, *n* = 16 with two AAI scores, 72% female, *M*_age_ = 18.72, *SD*_age_ = 1.07). Test-retest reliability for both of the established scales was quite high, MARS: α = 0.91, 95% CI: 0.72–0.97; STAI: α = 0.95, 95% CI: 0.87–1.0. The reliability for all the subscales of the AAI were also well above the α = 0.70 criterion for Cronbach's alpha (Peterson, 1994). The math (AAI-MATH: α = 0.94, 95% CI: 0.64–1.01) and writing subscales (AAI-WRI: α = 0.92, 95% CI: 0.75–1.01) were the most consistent over time. Test (AAI-TEST: α = 0.89, 95% CI: 0.69–0.98) and trait anxiety (AAI-TRAIT: α = 0.89, 95% CI: 0.56–1.0) were slightly less consistent across time. Science Anxiety (AAI-SCI: α = 0.83, 95% CI: 0.56–0.99) had the greatest variability across time, but still remained above the standard reliability coefficient of 0.70. Although this is a small sample of the undergraduate population, these reliability scores provide evidence that these scales have sufficient test-retest reliability.

To further assess reliability within these scales, we calculated the item-wise reliability for each scale and subscale to determine whether responses to the questions within each scale and subscale were sufficiently consistent. Reliability for both the MARS (α = 0.94, 95% CI: 0.93–0.96) and STAI (α = 0.93, 95% CI: 0.91–0.94) were very high, as would be expected by previous validation studies (Suinn and Winston, 2003). Reliability for the AAI subscales was slightly lower, but still above the 0.7 criterion. The AAI-Math subscale was the most reliable (α = 0.9, 95% CI: 0.87–0.92). All other subscales had reliability that was sufficiently high (AAI-Science: α = 0.83, 95% CI: 0.78–0.88; AAI-Writing: α = 0.84, 95% CI: 0.81–0.87; AAI-Test: α = 0.85 (95% CI: 0.82–0.87; AAI-Trait: α = 0.86, 95% CI: 0.81–0.90). While these alpha coefficients are not quite as high as observed in the online samples in Study 1, this slight drop in reliability is to be expected given that Study 1 specifically isolated and selected the responses to these items as the most similar (from the factor analysis and alpha coefficients), whereas in Study 2, we administered these questions as part of the AAI, a freestanding questionnaire. Overall, we observe that the subscales composing the AAI have a high degree of reliability.

#### Factor Analyses

We again used maximum-likelihood factor analysis to confirm the 5-factor structure hypothesized to represent the 5 domains of anxiety developed for the AAI. Factor loadings are reported in Table 5. Five factors sufficiently explain the variance among these items, *X*^2^_(985)_ = 1764.49, *p* < 0.001 [Tucker-Lewis Index of factoring reliability: 0.796; RMSEA index: 0.066 (95% CI: 0.055–0.064); BIC: −3561.42]. We also sought to determine how many items would be “correctly” classified in the latent factor structure, such that each item would have factor loadings that were sufficiently high within the hypothesized domain. While the suggested criterion for “significant” factor loading would be ~0.30 (Yong and Pearce, 2013), we used a conservative threshold of 0.50 for these factor loadings to more stringently determine which items loaded onto each factor, as this value better identifies items that only load onto a single factor (bolded items in Table 5). Even with this more conservative threshold, we find that the majority of items were correctly classified for each domain (Math: 90%, Science: 70%, Test: 70%, Trait: 80%, Writing: 80%). Overall, this factor analysis confirms that the 5-factor model originally created for the AAI by isolating items to test the constructs of math, science, writing, test, and trait anxiety is represented by the 50 items selected to compose this scale.

**Table 5:** Factor loadings from maximum-likelihood factor analysis for AAI items from Study 2 and Study 3.

		**Undergraduate sample (*****N=236)***					**High school sample (*N = 91)***				
**Item**	**Subscale**	***F1***	***F2***	***F3***	***F4***	***F5***	***F1***	***F2***	***F3***	***F4***	***F5***
AAI 07	MATH	**0.85**	0.06	−0.03	0.11	−0.05	**0.86**	0.06	0.19	0.05	−0.01
AAI 11	MATH	**0.83**	0.08	−0.06	0.15	−0.02	**0.80**	0.13	0.12	0.09	−0.01
AAI 12	MATH	**0.77**	0.09	0.02	0.11	0.05	**0.78**	0.18	0.05	−0.07	0.11
AAI 16	MATH	**0.71**	0.17	0.18	0.09	0.02	**0.67**	**0.52**	0.03	0.17	−0.08
AAI 26	MATH	0.24	−0.04	−0.03	0.41	0.03	0.44	0.14	0.09	0.12	−0.30
AAI 37	MATH	**0.51**	−0.05	0.03	0.35	0.32	**0.63**	−0.33	0.08	0.10	−0.04
AAI 40	MATH	**0.83**	0.00	0.05	0.05	−0.01	**0.89**	0.14	0.09	−0.06	−0.03
AAI 41	MATH	**0.74**	0.16	0.10	0.18	0.01	**0.71**	0.17	0.10	0.28	0.04
AAI 42	MATH	**0.64**	0.08	0.06	0.06	−0.01	**0.76**	0.17	0.17	−0.03	−0.02
AAI 48	MATH	**0.72**	0.12	0.06	0.16	−0.07	**0.57**	0.13	0.10	−0.13	−0.07
AAI 02	SCIENCE	0.08	−0.26	0.19	0.46	0.27	0.14	−0.61	0.12	0.38	0.13
AAI 06	SCIENCE	0.11	−0.03	0.11	**0.60**	0.06	0.19	−0.28	0.13	0.47	0.24
AAI 13	SCIENCE	0.27	−0.02	−0.01	**0.59**	0.04	0.27	0.09	0.02	**0.52**	−0.06
AAI 22	SCIENCE	0.19	0.18	0.15	**0.58**	0.11	−0.05	0.09	0.25	**0.56**	0.04
AAI 24	SCIENCE	−0.03	0.00	0.24	0.47	0.05	−0.04	−0.03	0.13	**0.55**	0.24
AAI 28	SCIENCE	0.20	0.08	0.11	**0.73**	0.14	0.07	−0.05	−0.07	**0.76**	−0.15
AAI 31	SCIENCE	0.08	0.07	0.13	**0.74**	0.07	0.05	0.10	0.06	**0.74**	0.06
AAI 32	SCIENCE	0.09	0.20	0.08	**0.77**	−0.01	−0.14	−0.06	0.17	**0.59**	−0.05
AAI 49	SCIENCE	−0.04	0.11	0.15	**0.70**	−0.07	0.10	0.22	0.01	**0.65**	−0.06
AAI 50	SCIENCE	0.11	−0.25	0.14	0.37	0.33	0.02	**−0.52**	0.09	0.18	0.14
AAI 03	TEST	0.05	−0.16	0.15	0.28	0.22	0.39	0.02	0.22	0.05	0.10
AAI 10	TEST	0.15	**0.72**	0.28	−0.04	0.02	0.16	**0.77**	0.32	0.15	0.06
AAI 19	TEST	0.20	**0.70**	0.13	0.00	0.00	0.33	**0.68**	0.23	0.21	0.05
AAI 21	TEST	0.00	**0.72**	0.11	0.00	0.10	0.13	**0.75**	0.18	0.03	−0.09
AAI 25	TEST	0.24	0.47	0.29	0.05	0.21	**0.53**	0.17	0.48	0.04	0.09
AAI 30	TEST	0.27	0.38	0.38	0.09	0.16	0.35	0.36	0.28	0.21	0.03
AAI 35	TEST	0.06	**0.68**	0.16	0.02	0.00	0.30	**0.71**	0.12	0.16	−0.01
AAI 44	TEST	0.07	**0.78**	0.15	−0.01	−0.08	0.30	**0.78**	0.25	0.06	0.04
AAI 46	TEST	0.03	**0.82**	0.14	0.01	−0.08	0.16	**0.76**	0.22	0.00	−0.03
AAI 47	TEST	0.13	**0.78**	0.08	0.11	0.06	0.19	**0.77**	0.12	0.10	−0.04
AAI 01	TRAIT	0.02	0.23	0.30	−0.09	−0.31	0.00	0.39	**0.65**	−0.01	−0.21
AAI 14	TRAIT	0.11	0.22	**0.64**	0.16	0.15	0.45	0.46	**0.51**	0.11	−0.01
AAI 15	TRAIT	0.04	0.22	**0.63**	0.13	−0.02	0.16	0.22	**0.68**	0.21	−0.03
AAI 17	TRAIT	0.03	0.10	**0.71**	0.06	0.15	0.28	0.06	**0.53**	0.13	−0.12
AAI 18	TRAIT	0.03	0.06	**0.70**	0.04	0.18	0.30	−0.15	**0.58**	0.16	0.15
AAI 20	TRAIT	0.07	0.33	**0.70**	0.15	−0.13	0.15	0.29	**0.74**	−0.01	0.13
AAI 23	TRAIT	−0.07	0.17	0.42	0.32	−0.02	−0.17	0.40	0.49	0.20	−0.08
AAI 29	TRAIT	0.07	0.16	**0.74**	0.14	0.07	0.20	0.18	**0.80**	0.15	−0.01
AAI 33	TRAIT	−0.02	0.02	**0.72**	0.24	0.13	0.16	−0.05	**0.78**	0.00	0.07
AAI 39	TRAIT	0.07	0.23	**0.54**	0.11	0.09	0.12	0.14	**0.64**	0.07	0.00
AAI 04	WRITING	−0.02	−0.04	0.05	0.00	**0.56**	0.09	−0.17	−0.05	−0.12	**0.71**
AAI 05	WRITING	0.02	−0.04	0.01	0.15	**0.67**	−0.07	−0.12	−0.10	0.02	**0.52**
AAI 08	WRITING	0.09	−0.08	0.37	0.08	**0.52**	0.25	0.06	0.32	0.22	**0.55**
AAI 09	WRITING	0.13	0.35	0.19	0.11	**0.55**	0.00	**0.56**	0.29	0.22	0.38
AAI 27	WRITING	−0.03	0.18	0.17	0.07	**0.60**	−0.13	0.22	−0.05	0.31	0.48
AAI 34	WRITING	0.09	0.10	0.44	0.27	0.43	0.25	0.28	0.17	0.38	0.43
AAI 36	WRITING	−0.12	0.12	−0.04	−0.01	**0.77**	−0.12	−0.08	−0.29	0.07	**0.68**
AAI 38	WRITING	0.00	0.02	0.00	−0.01	**0.78**	−0.03	−0.16	0.07	−0.14	**0.76**
AAI 43	WRITING	0.00	0.07	0.09	0.07	**0.72**	0.03	0.10	0.13	−0.03	**0.71**
AAI 45	WRITING	0.01	**0.54**	0.21	0.21	0.27	−0.08	0.30	0.30	0.34	0.43

*Factor loadings for Study 2 are presented in the columns on the left, Study 3 on the right. Factor loadings >0.5 depicted in bold to illustrate items that have high loadings on a particular factor*.

#### Correlations

One of the main goals in creating the AAI was to create subscales that reduced the degree of intercorrelation between domains of math, science, writing, test, and trait anxiety (Table 6). To test this, correlations were performed between these subscales and the STAI and MARS. The AAI-Math measures a similar construct to the MARS, as it is correlated with the MARS [*r*_(228)_ = 0.49, *p* < 0.001]. However, the AAI-Math scale provides a more unique measure of the construct of math anxiety, such that compared to the MARS, the AAI-Math subscale has reduced associations with all the other measures of anxiety. The STAI and AAI-Trait are also highly correlated, *r*_(228)_ = 0.80, *p* < 0.001. For AAI-Trait, the degree of intercorrelation with other measures is either reduced (in the case of AAI-Math) or comparable to what is found for the STAI (e.g., AAI-Science is slightly more strongly correlated with AAI-Trait than the STAI).

**Table 6:** Pearson correlations between domain subscales of the AAI and established measures of math and trait anxiety in Study 2.

**Undergraduate sample (*****N*** **=** **236)**							
	**STAI**	**MARS**	**AAI-MATH**	**AAI-SCI**	**AAI-WRI**	**AAI-TRAIT**	**AAI-TEST**
STAI	***–***	***0.421***	**0.162**	***0.347***	***0.299***	***0.859***	***0.511***
MARS		**–**	***0.487***	***0.428***	***0.300***	***0.412***	***0.537***
AAI-MATH			–	***0.314***	0.084	**0.149**	***0.245***
AAI-SCI				–	***0.307***	***0.388***	***0.190***
AAI-WRI					–	***0.342***	***0.285***
AAI-TRAIT						***–***	***0.491***
AAI-TEST							**–**

*Pearson correlations listed in bold are significant at α = 0.05, items significant at α = 0.01 are listed in bold italics*.

### Discussion

In Study 2, we sought to validate the AAI constructs that were developed from on the results of Study 1 by administering this scale, along with two other self-report scales of math and trait anxiety, to a sample of undergraduate students. Our goal was to determine whether the AAI reliably represented the constructs of math, science, writing, test, and trait anxiety, while reducing the overlap between these domains. In Study 2, we find that the AAI subscales have sufficient internal reliability, as well as test-retest reliability (though this latter analysis included only a small subsample of ~10% of participants). As in Study 1, the AAI subscales also reduce the degree of correlation between the domains, especially compared to the existing measures of the MARS and the STAI. As intended, the AAI-Math subscale was found to correlate significantly with the MARS, but was less related to all the other measures of anxiety than the MARS.

## Study 3

In Study 3, we examine the reliability of the AAI for a high school-aged adolescent sample. Further, we examine the criterion validity of the AAI by using it to predict deficits in a high school math class, and comparing results to those of other measures. Thus, we demonstrate that the math subscale of the AAI is a sensitive and specific measure of math anxiety.

### Method

#### Participants

Adolescent participants were recruited from a small, local high school in rural New England. Participants were enrolled as part of their participation in beginning and advanced algebra and geometry classes. All parents and guardians were provided with consent information and given the option to opt out of any study procedures, and all students included in the dataset provided verbal assent after being read an assent statement that reviewed the study procedures (2 students opted out). Written consent was not required because all study procedures were included as part of normal classroom procedures in collaboration with the school, and presented minimal risk to participants. Across these classes, 90 students were enrolled in the study, with nine students enrolled in multiple classes^1^ (*N* = 99 samples, 60% female, *M*_*age*_ = 15.34, *SD*_*age*_ = 1.05). Participants scoring >3 standard deviations from the mean on any of the questionnaires administered were removed from the relevant analyses as outliers. All study procedures were approved by the CPHS and the local school administration.

#### Procedures

At the beginning of the term, researchers were introduced to the class, and students were split into groups to learn about a variety of study techniques to improve math performance. Details of the study skills activity will be detailed in another work (Pizzie and Kraemer, 2017b). After this study skills activity, participants were asked to complete a variety of questionnaires about their experiences in math class, namely the AAI, as well as other, validated questionnaires to assess math anxiety: MARS (Suinn and Winston, 2003), test anxiety: TAI (Spielberger, 2009), and trait anxiety: STAI—trait subscale (Spielberger, 2010). Approximately 8 weeks later, students in each class completed a midterm examination, testing information recently learned as a part of their normal classroom activities. These numerical grades represent the percentage of correct answers on their classroom exam.

### Results

#### Reliability

As in Study 2, inter-item reliability was calculated for all the items within each subscale of the AAI (10 items each), as well as the established scales: MARS, TAI, and STAI. Reliability was high for the MARS (α = 0.95, 95% CI: 0.92–0.96), the TAI (α = 0.96, 95% CI: 0.95–0.97), and the STAI (α = 0.93, 95% CI: 0.90–0.94). Reliability was also sufficiently high for the AAI subscales: AAI-Math: α = 0.91, 95% CI: 0.87–0.94; AAI-Science: α = 0.78, 95% CI: 0.68–0.88; AAI-Writing: α = 0.81, 95% CI: 0.72–0.87; AAI-Test: α = 0.90, 95% CI: 0.85–0.93; AAI-Trait: α = 0.89, 95% CI: 0.83–0.95). Again, we observe that for the science subscale, the reliability is slightly lower than the other subscales, but it still above the 0.7 criterion (Peterson, 1994). Although the reliability coefficients for the validated scales (MARS, TAI, STAI) are slightly higher than what is observed for the AAI subscales, all measures are well above the suggested criterion for reliability.

#### Factor Analyses

Again, we evaluated the factor structure with a maximum-likelihood factor analysis using a varimax rotation, and also compared how this factor structure compared to the same analysis performed in Study 2 (Table 5). Although the sample size for this sample is substantially smaller than the original sample and may be slightly underpowered, here we use this analysis to show the consistency of the structure across samples. We find that 5 factors sufficiently explain the variance in responses on the AAI, *X*^2^_(985)_ = 1318.15, *p* < 0.001 [Tucker-Lewis Index of factoring reliability: 0.777; RMSEA index: 0.092 (95% CI: 0.052–0.07); BIC: −3114.1]. Using Pearson correlations, we compared the resulting factor structures from both Study 2 and Study 3. We find that across both samples, the resulting factor structures are highly correlated, *r*_(248)_ = 0.86, *p* < 0.001, 95% CI: 0.84–0.90, indicating that the factor loadings for these samples are consistent. As in Study 2, we find that the factor loadings for the items result in categorization in the hypothesized domain for the majority of the items (Math: 90%, Science: 70%, Test: 70%, Trait: 90%, Writing: 60%). Despite the fact that the factor loadings are slightly less consistent in this adolescent sample, given the consistency with the factor structure in Study 2, we can conclude that the across both undergraduate and adolescent samples, the five factor structure tests anxiety in the hypothesized domains of math, science, writing, test, and trait anxiety.

#### Correlations

We conducted Pearson correlations between each extant scale and the subscales of the AAI. Overall, the adolescent sample illustrates that the AAI subscales represent similar constructs as established measures of academic anxiety, while reducing the overlapping variance between these constructs, and measuring the unique aspects of academic anxiety in math, science, writing, test, and trait domains (Table 7). For example, the 20-item subscale of the STAI that measures trait anxiety correlates with the 10-item AAI-trait subscale at *r* = *0.8*6 in Study 2 and *r* = *0.8*9 in Study 3. More notably, the 30-item version of the MARS, which is commonly used to assess math anxiety, was again found to correlate strongly with test anxiety (*r* = 0.75), as measured by the TAI. As a further indication that the 10-item AAI-test subscale is assessing test anxiety, this subscale was strongly correlated with both the 20-item TAI (*r* = 0.87) and with the MARS (*r* = 0.79). As expected, the correlation between AAI-test and AAI-math was lower, though still positive (*r* = 0.55), indicating that the AAI subscales are strongly correlated with the longer scales from which they were derived, and yet are more separable between domains than these previously established measures.

**Table 7:** Pearson correlations between AAI subscales, established measures of math, trait and test anxiety, and midterm grades for Study 3.

**High school sample (*****N*** **=** **90)**									
	**GRADE**	**MARS**	**TAI**	**STAI**	**AAI-MATH**	**AAI-TEST**	**AAI-TRAIT**	**AAI-SCI**	**AAI-WRI**
GRADE	***–***	−0.004	−0.097	−0.028	**−0.252**	−0.002	0.028	−0.02	0.088
MARS		***–***	***0.748***	***0.600***	***0.531***	***0.785***	***0.573***	***0.298***	0.196
TAI			***–***	***0.652***	***0.550***	***0.866***	***0.664***	0.157	**0.235**
STAI				***–***	***0.455***	***0.599***	***0.893***	0.182	0.097
AAI-MATH					***–***	***0.545***	***0.422***	**0.231**	0.151
AAI-TEST						***–***	***0.595***	**0.207**	**0.245**
AAI-TRAIT							***–***	**0.292**	**0.213**
AAI-SCI								***–***	***0.288***
AAI-WRI									***–***

*Correlations listed in bold are significant at α = 0.05, items significant at α = 0.01 are listed in bold italics. Correlations with Grade represent Pearson correlations between the individual difference measure and percent accuracy on a midterm test in the students' mathematics class*.

In addition to validating the AAI subscales as reliable and accurate measures of anxiety in academic domains, we also utilized this sample in order to examine whether the AAI is predictive of deficits in a real-world setting. We conducted Pearson correlations between math test grades, the AAI subscales and the MARS, TAI, and STAI (Table 7). We hypothesized that math anxiety would predict lower exam scores on midterm exams administered in the classroom, and that these deficits should be uniquely associated with math anxiety or test anxiety, as these are the domain being tested. We find that the AAI-Math subscale was uniquely and significantly associated with lower performance on the midterm exam in mathematics classes, *r*_(94)_ = −0.252, *p* = 0.013; Table 7. No other individual difference measure, including the MARS and measures of test anxiety, was significantly correlated with test performance in math. In this way, we demonstrate the AAI-Math subscale is a sensitive and valid measure of deficits in performance attributed to math anxiety.

### Discussion

Across Studies 2 and 3, we confirmed the validity and reliability of the AAI subscales as separable measures of math, science, writing, test, and trait anxiety. Importantly, among this group of high school students, we find that the AAI-Math scale is a sensitive measure of deficits on a real world math examination. As in Study 2, in Study 3, we demonstrate that the AAI subscales had a high degree of reliability among the 10 items used for each subscale. Although the sample size may be slightly underpowered to make strong conclusions about the factor structure, that we find consistency with the original factor structure is a good confirmation that the five factor structure consistently depicts patterns of anxiety. We also demonstrate that while the AAI subscales are correlated with established measures of math, test, and trait anxiety, the AAI subscales testing these constructs represent less overlapping variance, as evidenced by reduced correlations between the AAI subscales. Overall, Study 3 demonstrates that the AAI is a reliable, sensitive, and valid measure of academic anxiety in high school students.

## Study 4

The results of Study 3 demonstrate that the AAI, particularly the AAI-Math subscale, is a valid measure of math anxiety in a test-taking situation where students encountered increased pressure to perform. In Study 4, we further illustrate that the AAI is predictive of attitudes toward mathematics when participants react to mathematical expressions without the pressure to perform. Here we evaluate how the AAI-Math subscale (and other subscales) relates to perceptions of mathematical complexity, estimations of accuracy, and emotionality while performing mathematics. We hypothesized that the AAI-Math subscale would be strongly predictive of negative perceptions of mathematical expressions, especially when the problems were difficult. We predict that other types of anxiety, and other scales measuring math anxiety would not be sensitive and specific enough to pick up on these item-level reactions to math stimuli.

### Method

#### Participants

Thirty-four undergraduate students were recruited to participate in Study 4. One participant was excluded for low accuracy/below-chance responding on an attention task that was not discussed in the present paper, for a final sample of 33 individuals between the ages of 18 and 27 years old, *M*_*age*_ = 19.64, *SD*_*age*_ = 2.13, 76% female. All participants provided signed written consent to participate in this research, and all procedures were approved by the CPHS.

#### Procedure

Participants were first asked to complete an attentional deployment task, the results of which are not germane to the present hypotheses and will be explored elsewhere. Participants were then asked to view and make ratings of mathematical expression stimuli. Stimuli were drawn from three categories: “Easy” stimuli were arithmetic problems that required knowledge of order of operations [for example, “1 + (2 – 3) ÷ 4”], “Moderate” stimuli included multiple terms, exponents, fractions, geometric and algebraic symbols [for example, “4*ln*(*x*) + 3*x*”], and “Hard” stimuli included more complex math drawn from linear algebra and calculus, emphasizing complex algebraic terms and Greek symbols [for example, “$xy=\;\int \frac{1}{x}dx$”]. Participants made ratings (1–7) of each stimulus (60 of each type, 180 stimuli total). Stimuli were rated on perceived complexity (“How complex is this stimulus?” 1: not at all, to 7: extremely), estimated ability to solve correctly (“If you had to solve this mathematical expression, how likely is it that you would get the correct answer?” 1: not at all, to 7: extremely), and emotionality (“Thinking about your emotions, if you had to work on this math problem, how would you feel?” 1: negative, to 7: positive). Participants were asked to rate all stimuli on one aspect at a time, and stimuli were presented in randomized order. Participants were not asked to solve any mathematical expression. After completing the rating task, participants were asked to complete questionnaires to determine their individual level of anxiety in various domains, including the AAI, as well as demographic information (same as in Study 3).

### Results

Scores for each scale were calculated by averaging responses. Ratings for each domain were calculated by averaging mean responses for the “Easy,” “Moderate,” and “Hard” problems. Outliers for any scale or category (>3 *SD* away from the mean) were removed from the dataset. Two participants' scores were reversed on the estimated accuracy ratings because it was believed that they had used the endpoints of the scale incorrectly (their ratings were inconsistent with the direction of the other ratings they had made).

Bivariate correlations were calculated between individual difference measures assessing math, science, writing, test, and trait anxiety and the ratings made on mathematical expressions. By correlating how individuals perceive individual mathematical expressions, and how these perceptions relate to math anxiety and anxiety in other domains, we illustrate that the AAI-Math subscale was not only predictive of deficits in performance of mathematics during an exam (Study 3), but also that the attitudes expressed in this self-report scale are consistent with ratings of individual mathematical expressions, giving us insight into perceptions about mathematics even when computations are not required.

Correlations between the ratings and anxiety scales are depicted in Table 8. The AAI-Math subscale predicts attitudes toward mathematical expressions regarding complexity, estimated accuracy, and negative emotionality. When these expressions are broken down into easy, moderate, and hard category, the AAI-Math subscale is associated with increased ratings of complexity for both moderate and hard expressions, is associated with decreased estimations of accuracy for moderate and hard expressions and more negative emotionality for moderate and hard problems. The AAI-Math did not predict attitudes in the easy expression category for any of these ratings, which is consistent with previous work showing impairment on more difficult problems compared to easy problems (Faust et al., 1996). The higher the score on the AAI-Math subscale, the more difficult the moderate and complex problems appear to be, the more individuals perceive that they are less likely to do these problems correctly, and the more negative affect they would experience if they had to solve them. These ratings illustrate that the AAI-Math subscale is predictive of ratings of individual math expressions that are consistent with overall negative attitudes and underestimation of mathematical ability independent of performance pressure as experienced during an exam.

**Table 8:** Correlations between anxiety measures and ratings of mathematical expressions in Study 4 (*N* = 33).

**Complexity**					**Estimated Accuracy**					**Emotionality**				
	**Easy**	**Moderate**	**Hard**	**All**		**Easy**	**Moderate**	**Hard**	**All**		**Easy**	**Moderate**	**Hard**	**All**
AAI MATH	−0.06	***0.52***	**0.44**	**0.45**	AAI MATH	−0.07	***−0.61***	***−0.57***	***−0.68***	AAI MATH	0.05	***−0.70***	***−0.63***	***−0.60***
AAI SCIENCE	−0.19	0.08	0.17	0.08	AAI SCIENCE	−0.07	−0.06	−0.12	−0.05	AAI SCIENCE	0.01	−0.03	−0.03	−0.02
AAI TEST	**0.37**	0.17	0.07	0.19	AAI TEST	−0.21	−0.18	−0.14	−0.06	AAI TEST	−0.06	−0.07	−0.001	−0.06
AAI TRAIT	0.08	0.27	0.31	0.27	AAI TRAIT	−0.005	**−0.42**	***−0.48***	***−0.52***	AAI TRAIT	−0.06	**−0.44**	**−0.54**	**−0.43**
AAI WRITING	−0.16	0.10	0.21	0.11	AAI WRITING	0.03	0.09	−0.10	−0.03	AAI WRITING	0.17	−0.06	−0.08	0.07
MARS	0.22	**0.40**	0.36	**0.40**	MARS	−0.21	**−0.40**	**−0.40**	**−0.36**	MARS	0.09	−0.29	−0.28	−0.23
STAI	0.12	**0.39**	0.33	0.36	STAI	−0.19	−0.33	−0.33	**−0.42**	STAI	0.02	−0.33	−0.26	−0.27
TAI	0.20	0.25	0.26	0.28	TAI	−0.31	−0.20	−0.31	**−0.39**	TAI	−0.06	−0.31	−0.31	−0.31

*Correlations listed in bold are significant at α = 0.05, items significant at α = 0.01 are listed in bold italics. Participants rated mathematical expressions on their perceived complexity (1: simple, 7: complex), estimated ability to do the problem correctly (1: unlikely, low estimated accuracy, 7: very likely, high estimated accuracy), and how they would feel if they were asked to work on that math problem (1: negative, 7: positive)*.

Comparing the AAI-Math subscale to the MARS, we find the MARS is not as strongly associated with attitudes regarding complexity, estimated accuracy and negative emotionality. Whereas, the AAI-Math subscale was associated with all three attitude domains, the MARS was associated with perceived complexity, mainly for moderate expressions, and estimate accuracy for moderate and hard expressions (and overall). However, the MARS was not associated with any ratings of emotionality for mathematical expressions. This difference between the MARS and the AAI-Math subscale may be indicative of the idea that although the MARS has been shown to be predictive of deficits in mathematics performance in the past (Hopko, 2003), the MARS may be more closely associated with aspects of complexity and performance under pressure (such as tests). In contrast, the AAI-Math subscale is additionally more sensitive to negative affect associated with math, as intended.

Other domains of anxiety are also significantly correlated with attitudes toward mathematics. The AAI-Trait subscale is associated with ratings of estimated accuracy, as well as negative emotionality associated with mathematics. Similarly, overall, the STAI and TAI are associated with estimated accuracy, indicating that broader patterns of test and trait anxiety are associated with attitudes that indicate reduced confidence in the ability to accurately perform mathematics.

### Discussion

Overall, the results of Study 4 provide convergent validity for the AAI-math subscale by illustrating that this measure is consistent with self-reported attitudes about complexity, estimated accuracy, and negative emotionality of given math expressions. Consistent with the intended purpose of the measure, this subscale is more sensitive to these attitudes than previously established measures, such as the MARS. Importantly, the AAI-Math subscale predicts negative emotion elicited by mathematics for challenging problems, and differences in these attitudes were not detected by the MARS. Moreover, the AAI-Math subscale is sensitive to these differences in perceptions of math problems, even when individuals are not asked to perform mathematical computations, which provides evidence that this scale is independent of the specific pressures of performance, such as those experienced during an exam.

## Overall Discussion

Across four studies, we demonstrate that the AAI, a self-report measure developed to measure anxiety in math, as well as contributions of anxiety associated with science, writing, test, and trait domains is a reliable and valid measure of these constructs across a wide population. We established the AAI using specific questions to reliably and independently assess anxiety associated with mathematics, as well as differentiating math anxiety from four other domains of anxiety: science, writing, test and trait anxiety. The AAI is easily administered by educators and researchers, and at 50 questions, represents a significant reduction in the time required to assess multiple domains of academic anxiety. The AAI-math subscale alone is only 10 items and predicts negative attitudes about math and math performance decrements as well as or better than any other existing measures. As a complete measure, the AAI isolates the items that are most uniquely predictive within each construct, creating a measure that maintains reliability, and has similar or better capacity to predict attitudes toward mathematics, as well as real-world classroom outcomes.

What is math anxiety? How do we uniquely measure math anxiety? Across four different studies, we demonstrate that math anxiety is a separable domain of anxiety from other measures of academic anxiety, and that by isolating specific questions regarding math anxiety, we can predict negative reactions toward mathematics, as well as performance deficits associated with math anxiety in a high school math classroom. The AAI-Math subscale is composed of questions that probe negative attitudes toward mathematics, as well as phrasing attitudes toward mathematics in a positive direction. That these attitudes and cognitive appraisals of mathematics are most diagnostic of the unique aspects of math anxiety is not surprising. Indeed, there may be many aspects of anxiety that can be shared across domains, such as physiological arousal, that individuals experience across math anxiety, test anxiety, etc. However, because these experiences of increased anxiety across domains are not unique to a specific domain, while they may be interesting, they may be less informative when trying to specifically understand the aspects of math anxiety that are unique. For example, it may not be sufficient to ask “how anxious” math anxious individuals feel while performing mathematics, because individuals who have a great deal of test anxiety or who generally feel anxious across a number of different situations may also endorse this statement, even though their anxiety is not specific to mathematics. While it is informative to understand what aspects of math anxiety are shared with other instances in which individuals can experience anxiety, in order to better understand how math anxiety is developed over the lifespan, the changes in cognition associated with math anxiety, and how best to remediate the deficits associated with increased anxiety, it may be most informative to highlight the aspects of math anxiety that are more unique and specific to the experience of math anxiety.

In Study 1, we used a large sample of adult participants to assess the validity and reliability of previously established questionnaires. Across two large samples of self-report data, we find that using just 10 items per domain adequately represents these domains of anxiety with a high degree of inter-item reliability within each subscale, while reducing the overlap between these constructs. Study 1 laid the groundwork for the AAI, selecting the specific questions that would be used to represent the 5 domains of anxiety, identifying items that uniquely represent math anxiety, as well as other facets of academic anxiety. Studies 2 and 3 evaluated the AAI as a freestanding 50-item questionnaire administered to undergraduate (Study 2) and adolescent (Study 3) populations. Study 4 demonstrates that the AAI-Math subscale is representative of reactions to mathematical expressions in low stakes environments. Across these studies, we demonstrated that the 10 items selected to represent each domain of the AAI are reliable measures of anxiety in these domains, and reduce the overlap between the domains compared to other previously established measures of math and trait anxiety, and are more sensitive and specific than previously established measures. In Study 4, the AAI Math subscale predicts negative emotional reactions to mathematical expressions, and is more sensitive to these attitudes than other measures of math anxiety. Importantly, the results of Study 3 also illustrate that the math subscale of the AAI is inversely correlated with math test performance in a real-world classroom setting.

The current study is not without limitations. Most of the previous literature has focused on identifying academic anxiety in school-aged participants, from elementary through undergraduate education (Hembree, 1990). However, Study 1, in which we established these constructs, utilized a wider range of ages, including an older population of participants, to assess anxiety in different domains. This population may have different patterns of anxiety when compared to the younger student populations investigated in prior research. However, addressing this issue directly, validated the AAI with samples of undergraduates (Study 2 and 4) and in a classroom setting with adolescent participants (Study 3). That these samples all converge to represent similar constructs across five domains of anxiety provides strong evidence that the AAI is a valid assessment for a wide variety of ages and populations. However, for all studies, the large majority of participants were middle and upper-middle class white individuals, and these samples were not racially or ethnically diverse enough to adequately represent the population of the U.S. Further research is needed to characterize the influence that anxiety associated with these academic domains may have over the course of the lifespan, and to understand how these constructs influence racially, economically, and socially diverse populations.

In conclusion, the present studies sought to analyze the relationship between the current measures of academic anxieties, and to identify items that uniquely assess these domains, ultimately creating our own measure of five domains of academic anxiety: the AAI. Here we present an abbreviated measure of math anxiety, which also reliably assesses anxiety associated with science, writing, test and trait anxiety. The AAI-Math subscale is reliably associated with negative attitudes and performance in the mathematics domain while reducing the overlap between math anxiety and other domains of anxiety.

While the AAI is optimized to represent math anxiety, the additional subscales of the AAI allow researchers to quickly and easily assess the contributions of other types of anxiety without administering further questionnaires. Moreover, all 5 domains of the AAI represent more separable constructs than have previously been validated in the past, giving confidence that the scores on the AAI represent the “pure” constructs of each type of anxiety, reducing the overlap between them. The present work demonstrates that by choosing the appropriate questions to assess anxiety associated with each domain, we can improve the methods by which we assess these negative attitudes and emotions associated with academic activities. By creating a questionnaire that establishes individual levels of anxiety in each subject using fewer questions, the AAI provides administrators, researchers and educators with an easily administered and ecologically valid tool to assess areas where possible performance deficits may occur across a number of different academic environments, ultimately identifying areas in which students may need extra support in order to reach their full potential.

## Author Contributions

RP was responsible for data collection, data analysis, and completed the first drafts of the manuscript. DK was responsible for oversight of data collection and analysis, and editing of the manuscript. DK and RP worked in collaboration on ideas, data analysis, and manuscript completion.

### Conflict of Interest Statement

The authors declare that the research was conducted in the absence of any commercial or financial relationships that could be construed as a potential conflict of interest.

## Footnotes

^1^Because a student's experience in one class was independent from their performance in the other class (i.e., one's performance in geometry is graded independently from performance in algebra class), students enrolled multiple classes were treated as independent subjects. Removal of secondary class data does not impact the results or conclusions explained in the results of this study.
