# Supplementary material for: The Academic Anxiety Inventory: Evidence for Dissociable Patterns of Anxiety Related to Math and Other Sources of Academic Stress
Source: Front Psychol. 2019 Jan 15;9:2684. doi: 10.3389/fpsyg.2018.02684 (PMC6340929; doi:10.3389/fpsyg.2018.02684)
Supplement: Supplementary file 1 [file Table_1.DOCX]

SUPPLEMENTARY INFORMATION

**Study 1**

**Results**

*Single Scales*

To assess the relationship between different types of anxiety, first we compared responses on the most commonly-used measure for each domain of anxiety. Primary measures for each domain were selected based on their prevalence in the literature, which was operationally defined by number of citations using Google Scholar (Table 1, Google Scholar). Each questionnaire was scored by calculating the average response for each scale or subscale (reverse-scored items were reversed before mean response was calculated). Bivariate correlations were used to explore the relationships between each questionnaire, and regression models were built for each domain: math, science, and writing anxiety (Table 2).

Math anxiety measured by the MARS was associated with scores in test anxiety (TAI) and science anxiety (SAQ-S; for beta values and estimates of variance explained, Supplementary Table 1). While adding additional factors such as trait anxiety resulted in slight increases in variance explained by these models, they were not significant improvements. The combination of science anxiety and test anxiety accounted for 55% of the variance in the original sample, and 59% of the variance in the test sample.

Science anxiety (as measured by the SAQ-S), in turn, was associated with math anxiety (MARS, Supplementary Table 2). The MARS accounted for comparable amounts of variance in both the original (41% of variance explained) and test (46% of variance explained) datasets. Adding additional factors to the MARS does not create significant improvements in the amount of variance explained across the original and test samples. Therefore, the regression model that contains only the MARS should be considered the best regression model associated with science anxiety.

Writing anxiety scores (measured by the WA) were best accounted for by test anxiety (TAI, Supplementary Table 3). The addition of additional regressors did not result in consistent improvements across the original and test sample. Moreover, it’s worth noting that the percentage of variance explained by other factors in the WA was much smaller than for the SAQ-S and the MARS, indicating that the WA is a relatively independent measure (i.e., little overlap between WA and anxiety in other domains).

These results using the popular measurements of math, science, and writing anxiety indicate a high degree of overlap between math, science, and test anxiety. While trait anxiety was a significant regressor for all of these factors, the explanatory variables that determined the largest amount of variance were associated with a specific domain of anxiety, rather than attributable to general patterns of anxiety or negative affect.

Supplementary Table 1. *Writing Anxiety, Trait Anxiety, Test Anxiety, and Science Anxiety as Predictors of Math Anxiety in Study 1*

|  |  | β | | | |  |
| --- | --- | --- | --- | --- | --- | --- |
| Model | Adj. R^2^ | WA | STAI | TAI | SAQ-S | |
| Original Dataset (*N* = 285) |  |  |  |  |  | |
| Model 1: WA | -.003 | -.03 |  |  |  | |
| Model 2: STAI | .11*** |  | .45*** |  |  | |
| Model 3: TAI | .37*** |  |  | .76*** |  | |
| Model 4: SAQ-S | .41*** |  |  |  | .79*** | |
| Model 5: SAQ-S + STAI | .43*** |  | .24*** |  | .73*** | |
| *Model 6: SAQ-S + TAI* | .55*** |  |  | .51*** | .57*** | |
| Model 7: SAQ-S + STAI + TAI | .55*** |  | .07 | .49*** | .56*** | |
| Test Dataset (*N* = 280) |  |  |  |  |  | |
| Model 1: WA | .04*** | -.19*** |  |  |  | |
| Model 2: STAI | .25*** |  | .61*** |  |  | |
| Model 3: TAI | .49*** |  |  | .82*** |  | |
| Model 4: SAQ-S | .46*** |  |  |  | .72*** | |
| Model 5: SAQ-S + STAI | .51*** |  | .31*** |  | .61*** | |
| *Model 6: SAQ-S + TAI* | .59*** |  |  | .53*** | .42*** | |
| Model 7: SAQ-S + STAI + TAI | .60*** |  | .16** | .47*** | .39*** | |

Note: The best regression model for MARS is depicted in italics. * *p* < .05, ** *p* < .01, *** *p* < .001

Supplementary Table 2. *Writing Anxiety, Trait Anxiety, Test Anxiety, and Math Anxiety as Predictors of Science Anxiety in Study 1*

|  |  | β | | | |  |
| --- | --- | --- | --- | --- | --- | --- |
| Model | Adj. R^2^ | WA | STAI | TAI | MARS | |
| Original Dataset (*N* = 285) |  |  |  |  |  | |
| Model 1: WA | .01* | -.09* |  |  |  | |
| Model 2: STAI | .07*** |  | .29*** |  |  | |
| Model 3: TAI | .19*** |  |  | .44*** |  | |
| *Model 4: MARS* | .41*** |  |  |  | .52*** | |
| Model 5: MARS + WA | .42*** | -.08* |  |  | .52*** | |
| Model 6: MARS + STAI | .41*** |  | .07 |  | .51*** | |
| Model 7: MARS + TAI | .41*** |  |  | .08 | .48*** | |
| Model 8: MARS + WA + TAI | 42*** | -.07* |  | .05 | .49*** | |
| Model 9: MARS + WA + STAI | .42*** | -.07* | .05 |  | .50*** | |
| Model 10: MARS + STAI + TAI | .41*** |  | .06 | .05 | .48*** | |
| Model 11: MARS + WA + STAI + TAI | .42*** | -.06 | .04 | .04 | .49*** | |
| Test Dataset (*N* = 280) |  |  |  |  |  | |
| Model 1: WA | .05*** | -.20* |  |  |  | |
| Model 2: STAI | .19*** |  | .50*** |  |  | |
| Model 3: TAI | .38*** |  |  | .67*** |  | |
| *Model 4: MARS* | .46*** |  |  |  | .64*** | |
| Model 5: MARS + WA | .42*** | -.08* |  |  | .63*** | |
| Model 6: MARS + STAI | .47*** |  | .15* |  | .59*** | |
| Model 7: MARS + TAI | .46*** |  |  | .30*** | .46*** | |
| Model 8: MARS + WA + TAI | 50*** | -.04 |  | .28*** | .46*** | |
| Model 9: MARS + WA + STAI | .42*** | -.07 | .13* |  | .57*** | |
| Model 10: MARS + STAI + TAI | .50*** |  | .09 | .27*** | .44*** | |
| Model 11: MARS + WA + STAI + TAI | .50*** | -.04 | .08 | .26*** | .44*** | |

Note: The best regression model for SAQ-S is depicted in italics. * *p* < .05, ** *p* < .01, *** *p* < .001

Supplementary Table 3. *Math Anxiety, Science Anxiety, Trait Anxiety, and Test Anxiety as Predictors of Writing Anxiety in Study 1*

|  |  | β | | | |  |
| --- | --- | --- | --- | --- | --- | --- |
| Model | Adj. R^2^ | SAQ-S | STAI | TAI | MARS | |
| Original Dataset (*N* = 285) |  |  |  |  |  | |
| Model 1: MARS | -.003 |  |  |  | -.04 | |
| Model 2: SAQ-S | .01*** | -.17*** |  |  |  | |
| Model 3: STAI | .03*** |  | -.24*** |  |  | |
| *Model 4: TAI* | .04*** |  |  | -.26*** |  | |
| Model 5: SAQ-S + STAI | .03*** | -.11 | -.22* |  |  | |
| Model 6: SAQ-S + TAI | .04*** | -.06 |  | -.24** |  | |
| Model 7: STAI + TAI | .04*** |  | -.16 | -.21* |  | |
| Model 8: SAQ-S + STAI + TAI | 04*** | -.04 | -.15 | -.19* |  | |
| Test Dataset (*N* = 280) |  |  |  |  |  | |
| Model 1: MARS | .04*** |  |  |  | -.22*** | |
| Model 2: SAQ-S | .05*** | -.26*** |  |  |  | |
| Model 3: STAI | .06*** |  | -.35*** |  |  | |
| *Model 4: TAI* | .09*** |  |  | -.37*** |  | |
| Model 5: SAQ-S + STAI | .08*** | -.17* | -.26*** |  |  | |
| Model 6: SAQ-S + TAI | .09*** | -.09 | -.31*** |  |  | |
| Model 7: STAI + TAI | .10*** |  | -.20* | -.28*** |  | |
| Model 8: SAQ-S + STAI + TAI | .10*** | -.05 | -.19* | -.25** |  | |

Note: The best model for WA is depicted in italics. * *p* < .05, ** *p* < .01, *** *p* < .001
