# Supplementary material for: The Academic Anxiety Inventory: Evidence for Dissociable Patterns of Anxiety Related to Math and Other Sources of Academic Stress
Source: Front Psychol. 2019 Jan 15;9:2684. doi: 10.3389/fpsyg.2018.02684 (PMC6340929; doi:10.3389/fpsyg.2018.02684)
Supplement: Supplementary file 2 [file Data_Sheet_1.pdf]

APPENDIX

AAI Scoring Guide and Academic Anxiety Inventory

ACADEMIC ANXIETY INVENTORY SCORING GUIDE

Please use the following to help score the AAI.

AAI Instructions: The following statements describe different feelings and situations, especially those you might encounter in school. Please read each item carefully and think about how it might apply to you. For each of the following statements, please choose the rating that best reflects your attitude in general:

STRONGLY DISAGREE	DISAGREE	NEUTRAL	AGREE	STRONGLY AGREE
1	2	3	4	5

SUBSCALE	ITEM	REVERSED
TRAIT	Sometimes important thoughts run through my mind and bother me.	
SCIENCE	Studying mathematics is just as appropriate for women as for men.	*
TEST	Reviewing study materials the night before an exam makes me feel confident.	*
WRITING	I like seeing my thoughts typed on screen or written on paper.	*
WRITING	Expressing ideas through writing seems to be a waste of time.	
SCIENCE	I feel confident I could cool down a hot tub of water to an appropriate temperature for a bath.	*
MATH	Mathematics is enjoyable and stimulating to me.	*
WRITING	Handing in an essay makes me feel good.	*
WRITING	I don't like my essays to be evaluated.	
TEST	I feel anxious while studying for a final.	
MATH	Figuring out mathematical problems does not appeal to me.	
MATH	I think I could handle more difficult mathematics.	*
SCIENCE	Adding 976 + 777 on paper would make me feel nervous.	
TRAIT	Too often, when things go wrong, I get discouraged and feel like giving up.	
TRAIT	I often feel like crying.	
MATH	For some reason even though I study, math seems unusually hard for me.	
TRAIT	I am not a cheerful optimist.	
TRAIT	I am a cheerful, high-spirited person.	*
TEST	I feel nervous studying for a test the night before.	
TRAIT	I wish I could be as happy as others seem to be.	
TEST	I feel anxious while waiting to see my letter grade on a test.	
SCIENCE	I would feel stressed reading a cash register receipt.	
TRAIT	I am losing out on things because I can't make up my mind soon enough.	
SCIENCE	I feel confident I could fill my bicycle tires with the right amount of air.	*

TEST	I feel confident in class waiting for my graded test to be returned.	*
MATH	Mathematics is less important to people than art or literature.	
WRITING	I have a terrible time organizing my ideas in a writing course.	
SCIENCE	Using a thermometer in order to record the boiling point of a heating solution makes me nervous.	
TRAIT	Sometimes I feel completely worthless.	
TEST	I feel confident while studying for a midterm.	*
SCIENCE	Focusing the lens on my camera makes me nervous about using the equipment.	
SCIENCE	I feel stressed trying to focus a microscope.	
TRAIT	I generally feel pleasant.	*
WRITING	When I hand in an essay, I know I'm going do to poorly.	
TEST	I feel nervous waiting for a test to be handed out.	
WRITING	Writing is a lot of fun.	*
MATH	Mathematics helps develop a person's mind and teaches him/her to think.	*
WRITING	I look forward to writing down my ideas.	*
TRAIT	I usually feel rested.	*
MATH	Mathematics is very interesting, and I have usually enjoyed courses in this subject.	*
MATH	I'm not good at math.	
MATH	I am sure I could do advanced work in mathematics.	*
WRITING	Discussing my writing with others is enjoyable.	*
TEST	I feel stressed thinking about a coming exam an hour before it's scheduled to begin.	
WRITING	I am afraid of writing essays when I know they will be evaluated.	
TEST	Thinking about a coming exam the night before its scheduled date makes me nervous.	
TEST	I feel anxious having a test returned.	
MATH	I do as little math a possible.	
SCIENCE	Mixing boiling water and ice to get water at 70 degrees Fahrenheit makes me nervous about getting the water to the right temperature.	
SCIENCE	If I wanted to vote on an upcoming referendum on student activities fees, I would feel more confident after reading about it so that I might make an informed choice.	*

Note: Higher scores represent more negative attitudes. Items with an asterisk in the “Reversed” column represent more positive attitudes and should be reverse scored in order to represent negative attitudes. That these items should be reversed should not be indicated to participants when the questionnaire is administered.

INSTRUCTIONS:

The following statements describe different feelings and situations, especially those you might encounter in school. Please read each item carefully and think about how it might apply to you. For each of the following statements, please choose (circle) the rating that best reflects your attitude in general:

Sometimes important thoughts run through my mind and bother me.

STRONGLY DISAGREE	DISAGREE	NEUTRAL	AGREE	STRONGLY AGREE
1	2	3	4	5

Studying mathematics is just as appropriate for women as for men.

STRONGLY DISAGREE	DISAGREE	NEUTRAL	AGREE	STRONGLY AGREE
1	2	3	4	5

Reviewing study materials the night before an exam makes me feel confident.

STRONGLY DISAGREE	DISAGREE	NEUTRAL	AGREE	STRONGLY AGREE
1	2	3	4	5

I like seeing my thoughts typed on screen or written on paper.

STRONGLY DISAGREE	DISAGREE	NEUTRAL	AGREE	STRONGLY AGREE
1	2	3	4	5

Expressing ideas through writing seems to be a waste of time.

STRONGLY DISAGREE	DISAGREE	NEUTRAL	AGREE	STRONGLY AGREE
1	2	3	4	5

I feel confident I could cool down a hot tub of water to an appropriate temperature for a bath.

STRONGLY DISAGREE	DISAGREE	NEUTRAL	AGREE	STRONGLY AGREE
1	2	3	4	5

Mathematics is enjoyable and stimulating to me.

STRONGLY DISAGREE	DISAGREE	NEUTRAL	AGREE	STRONGLY AGREE
1	2	3	4	5

Handing in an essay makes me feel good.

STRONGLY DISAGREE	DISAGREE	NEUTRAL	AGREE	STRONGLY AGREE
1	2	3	4	5

I don't like my essays to be evaluated.

STRONGLY DISAGREE	DISAGREE	NEUTRAL	AGREE	STRONGLY AGREE
1	2	3	4	5

I feel anxious while studying for a final.

STRONGLY DISAGREE	DISAGREE	NEUTRAL	AGREE	STRONGLY AGREE
1	2	3	4	5

Figuring out mathematical problems does not appeal to me.

STRONGLY DISAGREE	DISAGREE	NEUTRAL	AGREE	STRONGLY AGREE
1	2	3	4	5

I think I could handle more difficult mathematics.

STRONGLY DISAGREE	DISAGREE	NEUTRAL	AGREE	STRONGLY AGREE
1	2	3	4	5

Adding 976 + 777 on paper would make me feel nervous.

STRONGLY DISAGREE	DISAGREE	NEUTRAL	AGREE	STRONGLY AGREE
1	2	3	4	5

Too often, when things go wrong, I get discouraged and feel like giving up.

STRONGLY DISAGREE	DISAGREE	NEUTRAL	AGREE	STRONGLY AGREE
1	2	3	4	5

I often feel like crying.

STRONGLY DISAGREE	DISAGREE	NEUTRAL	AGREE	STRONGLY AGREE
1	2	3	4	5

For some reason even though I study, math seems unusually hard for me.

STRONGLY DISAGREE	DISAGREE	NEUTRAL	AGREE	STRONGLY AGREE
1	2	3	4	5

I am not a cheerful optimist.

STRONGLY DISAGREE	DISAGREE	NEUTRAL	AGREE	STRONGLY AGREE
1	2	3	4	5

I am a cheerful, high-spirited person.

STRONGLY DISAGREE	DISAGREE	NEUTRAL	AGREE	STRONGLY AGREE
1	2	3	4	5

I feel nervous studying for a test the night before.

STRONGLY DISAGREE	DISAGREE	NEUTRAL	AGREE	STRONGLY AGREE
1	2	3	4	5

I wish I could be as happy as others seem to be.

STRONGLY DISAGREE	DISAGREE	NEUTRAL	AGREE	STRONGLY AGREE
1	2	3	4	5

I feel anxious while waiting to see my letter grade on a test.

STRONGLY DISAGREE	DISAGREE	NEUTRAL	AGREE	STRONGLY AGREE
1	2	3	4	5

I would feel stressed reading a cash register receipt.

STRONGLY DISAGREE	DISAGREE	NEUTRAL	AGREE	STRONGLY AGREE
1	2	3	4	5

I am losing out on things because I can't make up my mind soon enough.

STRONGLY DISAGREE	DISAGREE	NEUTRAL	AGREE	STRONGLY AGREE
1	2	3	4	5

I feel confident I could fill my bicycle tires with the right amount of air.

STRONGLY DISAGREE	DISAGREE	NEUTRAL	AGREE	STRONGLY AGREE
1	2	3	4	5

I feel confident in class waiting for my graded test to be returned.

STRONGLY DISAGREE	DISAGREE	NEUTRAL	AGREE	STRONGLY AGREE
1	2	3	4	5

Mathematics is less important to people than art or literature.

STRONGLY DISAGREE	DISAGREE	NEUTRAL	AGREE	STRONGLY AGREE
1	2	3	4	5

I have a terrible time organizing my ideas in a writing course.

STRONGLY DISAGREE	DISAGREE	NEUTRAL	AGREE	STRONGLY AGREE
1	2	3	4	5

Using a thermometer in order to record the boiling point of a heating solution makes me nervous.

STRONGLY DISAGREE	DISAGREE	NEUTRAL	AGREE	STRONGLY AGREE
1	2	3	4	5

Sometimes I feel completely worthless.

STRONGLY DISAGREE	DISAGREE	NEUTRAL	AGREE	STRONGLY AGREE
1	2	3	4	5

I feel confident while studying for a midterm.

STRONGLY DISAGREE	DISAGREE	NEUTRAL	AGREE	STRONGLY AGREE
1	2	3	4	5

Focusing the lens on my camera makes me nervous about using the equipment.

STRONGLY DISAGREE	DISAGREE	NEUTRAL	AGREE	STRONGLY AGREE
1	2	3	4	5

I feel stressed trying to focusing a microscope.

STRONGLY DISAGREE	DISAGREE	NEUTRAL	AGREE	STRONGLY AGREE
1	2	3	4	5

I generally feel pleasant.

STRONGLY DISAGREE	DISAGREE	NEUTRAL	AGREE	STRONGLY AGREE
1	2	3	4	5

When I hand in an essay, I know I'm going do to poorly.

STRONGLY DISAGREE	DISAGREE	NEUTRAL	AGREE	STRONGLY AGREE
1	2	3	4	5

I feel nervous waiting for a test to be handed out.

STRONGLY DISAGREE	DISAGREE	NEUTRAL	AGREE	STRONGLY AGREE
1	2	3	4	5

Writing is a lot of fun.

STRONGLY DISAGREE	DISAGREE	NEUTRAL	AGREE	STRONGLY AGREE
1	2	3	4	5

Mathematics helps develop a person's mind and teaches him/her to think.

STRONGLY DISAGREE	DISAGREE	NEUTRAL	AGREE	STRONGLY AGREE
1	2	3	4	5

I look forward to writing down my ideas.

STRONGLY DISAGREE	DISAGREE	NEUTRAL	AGREE	STRONGLY AGREE
1	2	3	4	5

I usually feel rested.

STRONGLY DISAGREE	DISAGREE	NEUTRAL	AGREE	STRONGLY AGREE
1	2	3	4	5

Mathematics is very interesting, and I have usually enjoyed courses in this subject.

STRONGLY DISAGREE	DISAGREE	NEUTRAL	AGREE	STRONGLY AGREE
1	2	3	4	5

I'm not good at math.

STRONGLY DISAGREE	DISAGREE	NEUTRAL	AGREE	STRONGLY AGREE
1	2	3	4	5

I am sure I could do advanced work in mathematics.

STRONGLY DISAGREE	DISAGREE	NEUTRAL	AGREE	STRONGLY AGREE
1	2	3	4	5

Discussing my writing with others is enjoyable.

STRONGLY DISAGREE	DISAGREE	NEUTRAL	AGREE	STRONGLY AGREE
1	2	3	4	5

I feel stressed thinking about a coming exam an hour before it's scheduled to begin.

STRONGLY DISAGREE	DISAGREE	NEUTRAL	AGREE	STRONGLY AGREE
1	2	3	4	5

I am afraid of writing essays when I know they will be evaluated.

STRONGLY DISAGREE	DISAGREE	NEUTRAL	AGREE	STRONGLY AGREE
1	2	3	4	5

Thinking about a coming exam the night before its scheduled date makes me nervous.

STRONGLY DISAGREE	DISAGREE	NEUTRAL	AGREE	STRONGLY AGREE
1	2	3	4	5

I feel anxious having a test returned.

STRONGLY DISAGREE	DISAGREE	NEUTRAL	AGREE	STRONGLY AGREE
1	2	3	4	5

I do as little math a possible.

STRONGLY DISAGREE	DISAGREE	NEUTRAL	AGREE	STRONGLY AGREE
1	2	3	4	5

Mixing boiling water and ice to get water at 70 degrees Fahrenheit makes me nervous about getting the water to the right temperature.

STRONGLY DISAGREE	DISAGREE	NEUTRAL	AGREE	STRONGLY AGREE
1	2	3	4	5

If I wanted to vote on an upcoming referendum on student activities fees, I would feel more confident after reading about it so that I might make an informed choice.

STRONGLY DISAGREE	DISAGREE	NEUTRAL	AGREE	STRONGLY AGREE
1	2	3	4	5
